# The Impact of Single-Stranded DNA-Binding Protein SSB and Putative SSB-Interacting Proteins on Genome Integrity in the Thermophilic Crenarchaeon *Sulfolobus acidocaldarius*

**DOI:** 10.3390/ijms24054558

**Published:** 2023-02-25

**Authors:** Shoji Suzuki, Norio Kurosawa

**Affiliations:** 1 Super-Cutting-Edge Grand and Advanced Research (SUGAR) Program, Institute for Extra-Cutting-Edge Science and Technology Avant-Garde Research (X-Star), Japan Agency for Marine-Earth Science and Technology (JAMSTEC), 2-15 Natsushima-cho, Yokosuka 237-0061, Japan; 2 Department of Science and Engineering for Sustainable Innovation, Faculty of Science and Engineering, Soka University, Hachioji 192-8577, Japan

**Keywords:** hyperthermophilic archaea, mutation avoidance, homologous recombination, DNA repair, *Sulfolobus acidocaldarius*

## Abstract

The study of DNA repair in hyperthermophiles has the potential to elucidate the mechanisms of genome integrity maintenance systems under extreme conditions. Previous biochemical studies have suggested that the single-stranded DNA-binding protein (SSB) from the hyperthermophilic crenarchaeon *Sulfolobus* is involved in the maintenance of genome integrity, namely, in mutation avoidance, homologous recombination (HR), and the repair of helix-distorting DNA lesions. However, no genetic study has been reported that elucidates whether SSB actually maintains genome integrity in *Sulfolobus* in vivo. Here, we characterized mutant phenotypes of the *ssb*-deleted strain Δ*ssb* in the thermophilic crenarchaeon *S. acidocaldarius*. Notably, an increase (29-fold) in mutation rate and a defect in HR frequency was observed in Δ*ssb*, indicating that SSB was involved in mutation avoidance and HR in vivo. We characterized the sensitivities of Δ*ssb*, in parallel with putative SSB-interacting protein-encoding gene-deleted strains, to DNA-damaging agents. The results showed that not only Δ*ssb* but also Δ*alhr1* and ΔSaci_0790 were markedly sensitive to a wide variety of helix-distorting DNA-damaging agents, indicating that SSB, a novel helicase *Sac*aLhr1, and a hypothetical protein Saci_0790, were involved in the repair of helix-distorting DNA lesions. This study expands our knowledge of the impact of SSB on genome integrity and identifies novel and key proteins for genome integrity in hyperthermophilic archaea in vivo.

## 1. Introduction

Genomic DNA, which encodes genetic information, is continually damaged by endogenous and exogenous factors, and the frequency of this damage is accelerated by two to three orders of magnitude at high temperatures [1]. Hyperthermophiles are heat-loving microorganisms that flourish in hot environments (above 80 °C) [2]. The intriguing question of how hyperthermophiles consistently maintain their genome integrity under extreme environments has been discussed [1,3,4,5,6], and the idea that hyperthermophiles efficiently repair DNA damage that occurs at elevated levels has been proposed [1,3,4,5,6]. Thus, studies to elucidate DNA repair mechanisms in hyperthermophiles are important for understanding the broader mechanisms underlying the maintenance of genetic information in living cells under hot environments. Notably, most hyperthermophiles belong to the Archaea domain [2]; thus, the DNA repair mechanisms in hyperthermophilic archaea (HA) have been extensively studied [4,6,7,8,9,10,11,12]. However, these mechanisms still remain unclear, and several questions have yet to be answered [6,11,13].

The nucleotide excision repair (NER) pathway removes a wide variety of helix-distorting DNA lesions, such as UV-induced DNA damage (photoproducts), intrastrand crosslinks, and bulky adducts [6,11,13]. The NER process is generally composed of three steps, namely, the detection of DNA damage, unwinding of the double-stranded DNA (dsDNA) region by helicases, and incision by endonucleases [14]. HA have homologs of eukaryotic NER proteins, including the helicases XPB and XPD and the endonucleases XPF/Hef (C-terminal domain of euryarchaeal Hef is similar to XPF) and XPD [6,11,14]; however, HA lacks homologs of the NER damage recognition proteins XPC and XPA [6,14]. In addition, HA does not appear to use these proteins in NER, except for XPF/Hef [6,11,15,16,17]. Although the NER pathway in HA has not been identified, homologous recombination (HR)-mediated stalled-fork DNA repair has been proposed as a possible pathway for the repair of DNA helix distortion [6,15,17]. In this HR-mediated DNA repair process, it is hypothesized that the stalled replication fork at the helix-distorting DNA damage site is cleaved by flap endonucleases, and the lesion in the cleaved strand is removed by end resection. Finally, the replication fork is reassembled through HR [6]. Based on the results of genetic and biochemical studies of the 3′-flap endonuclease XPF/Hef [6,15,16,17,18,19,20] and NucS, which has flap endonuclease activity [17,21], it has been proposed that XPF/Hef and NucS are involved in HR-mediated stalled-fork DNA repair. However, the DNA repair process for helix-distorting DNA lesions in HA remains to be completely elucidated, and further analysis is needed.

In the case of the mismatch repair (MMR) pathway, which removes DNA replication errors, HA lacks the MutS-MutL-based canonical MMR system. Instead, HA have the mismatch-specific endonuclease EndoMS (another name for NucS) [22] and EndoMS/NucS, which are involved in mutation avoidance in the hyperthermophilic crenarchaeon *Saccharolobus* (formerly *Sulfolobus*) *islandicus* [23]. However, our genetic analysis in a previous study did not demonstrate that EndoMS/NucS was involved in mutation avoidance in the thermophilic crenarchaeon *Sulfolobus acidocaldarius*, which, similar to *Saccharolobus*, belongs to the order *Sulfolobales* [17]. For this reason, the mutation avoidance mechanism in *S. acidocaldarius* remains unclear.

Single-stranded DNA (ssDNA)-binding proteins, designated SSB in the bacteria and crenarchaea or replication protein A (RPA) in Eukaryotes and euryarchaea, specifically bind to ssDNA without sequence specificity via the oligonucleotide-binding fold (OB-fold) [24,25,26,27]. Canonical OB-fold SSB proteins are universally distributed in cellular organisms with some exceptions [28] and play essential roles in DNA replication, recombination, and repair [27,29,30,31]. These proteins are generally known to be involved in HR in cellular processes, and the functional mechanism is considered to entail binding to the ssDNA region of 3′-overhang DNA produced by the end resection process and protecting the formation of a secondary structure of ssDNA, resulting in the promotion of strand exchange, which is catalyzed by the recombinase in vitro [32,33,34,35]. In addition to HR, it has been proposed that SSB is also involved in DNA repair in HA. The *Sa. solfataricus* SSB can melt dsDNA containing a mismatched base or DNA lesions, such as a bulky adduct and cyclobutane pyrimidine dimer (CPD), in vitro [36], suggesting that SSB acts as not only a mismatched base but also a helix-distorting DNA damage detection protein in the order *Sulfolobales*. For these reasons, multiple intriguing SSB in vivo roles in the HA genome integrity has been hypothesized. However, there is no genetic evidence to show the involvement of SSB in HR and DNA repair in HA in vivo.

Previously, we succeeded in isolating the *ssb*-deleted strain of *S. acidocaldarius* [37]; however, that study reported only the growth phenotype regarding growth temperature. Further phenotypic characterization of the *ssb*-deleted strain was necessary to provide the genetic evidence described above. In addition, we previously identified a novel helicase—archaeal long helicase related (aLhr) 1, *Sac*aLhr1, in *S. acidocaldarius*—that dissociated a synthetic Holliday junction (HJ) in vitro [38]. Notably, the HR frequency in the *alhr1*-deleted strain is five-fold lower than that in the parent strain, indicating that *Sac*aLhr1 may be involved in HR in vivo [38]. However, its physiological role in DNA repair needs to be further characterized. The homolog of *Sac*aLhr1 was originally reported as a candidate protein interacting with an ssDNA–SSB complex in a biochemical study on SSB from *Sa*. *solfataricus* [36]. In addition, the pull-down experiment that Cubeddu and White conducted demonstrated that two unknown proteins with a helicase-like sequence and three hypothetical proteins were also copurified with the ssDNA–SSB complex [36], suggesting that these proteins interacted with SSB. Because the roles of SSB and *Sac*aLhr1 were of interest in the investigation of *S. acidocaldarius* genome integrity, we also focused on the roles of other putative SSB-interacting proteins described above.

Here, to explore the in vivo roles of SSB in genome integrity, namely, in HR, mutation avoidance, and NER, we characterized the phenotypes of the *ssb*-deleted strain of *S. acidocaldarius*, including phenotypes for mutation rate, HR frequency, sensitivity to DNA damage, and the capacity for the repair of UV-induced DNA damage (specifically, damage to CPDs). Moreover, in addition to investigating the *alhr1*-deleted strain, we constructed four gene-deleted strains that encoded unknown proteins with a helicase-like sequence and a hypothetical protein as candidates for SSB-interacting proteins on the basis of a previous report [36] and investigated the role of these proteins in genome integrity.

## 2. Results

### 2.1. Construction of Gene-Deletion Strains

To investigate the in vivo roles of SSB in DNA repair and HR in *S. acidocaldarius*, we decided to conduct a genetic study on SSB and five candidates for putative SSB-interacting proteins. Previously, Cubeddu and White [36] reported some candidate proteins interacting with the ssDNA–SSB complex in their study on *Sa. solfataricus* SSB. Three SF2 helicases, SSO0017, SSO0394, and SSO0965, and three hypothetical proteins, SSO0191, SSO1331, and SSO2452, were included in the candidate proteins. Comprehensive phylogenetic analysis of the SF2 and aLhr helicases in living things indicated that SSO0017, SSO0394, and SSO0965 were divided into SftH, aLhr1, and aLhr3, respectively [39,40], but no research on SftH and aLhr3 homologs were reported for Archaea. We recently characterized *Sac*aLhr1 helicase (Saci_0814) as an SSO0394 homolog in *S. acidocaldarius* [38], but it is not clear whether *Sac*aLhr1 is functionally required for DNA repair. Regarding the three hypothetical proteins, McRobbie et al. reported that the SSO2452 homolog was a recombination protein RecA paralog (Rad55) [41], and genetic analysis suggested that Rad55 (RadC1 encoded by SiRe_0240) was involved in DNA repair [42]. Thus far, no studies on SSO0191 and SSO1331 homologs have been reported. Notably, in the present study, *sftH* (Saci_0281, SACI_RS01370), *alhr3* (Saci_1320, SACI_RS06300), Saci_0790 (SACI_RS03780), and *rad55* (Saci_0546, SACI_RS02605) were found to share 60%, 53%, 46%, and 79% sequence identity with SSO0017, SSO0965, SSO0191, and SSO2454 over the entire amino acid sequence. However, no SSO1331 homolog was identified in the *S. acidocaldarius* genome. In this study, in addition to the *ssb* and *alhr1* genes, we decided to independently construct and genetically characterize four deletion strains: *sftH*, *alhr3*, Saci_0790, and *rad55*.

In our previous research, we constructed two deletion mutant strains of *S. acidocaldarius* for the gene encoding of SSB and *Sac*aLhr1 from the parental strain DP-1 [37,38]. Using the gene knockout strategy from our past work [43], we constructed *sftH*-, *alhr3*-, Saci_0790-, and *rad55*-deletion strains by deleting each gene coding region from the parental strain DP-1 (Figure S1A). The isolated strains were subjected to PCR using primers that were designed for the outer regions of target genes to confirm the deletion of the target gene from the original locus. The shortened PCR products were obtained using the outer primers from the genomic DNA of each isolated strain (Figure S1B–D). These results indicate that each gene was removed from the original locus of the genome of the knockout strains. The *rad55*-, *alhr3*-, Saci_0790-, and *sftH*-deletion strains were designated, *S. acidocaldarius* strains DP-13 (Δ*pyrE* Δ*suaI* Δ*phr* Δ*rad55*), DP-14 (Δ*pyrE* Δ*suaI* Δ*phr* Δ*alhr3*), DP-16 (Δ*pyrE* Δ*suaI* Δ*phr* ΔSaci_0790), and DP-18 (Δ*pyrE* Δ*suaI* Δ*phr* Δ*sftH*).

### 2.2. SSB Is Required for Mutation Avoidance in S. acidocaldarius

We studied the mutation frequency of Δ*ssb*; however, no obvious difference was observed between the mutation rate of Δ*ssb* and DP-1 when cells that were pre-cultivated at 75 °C in a liquid medium were used (7.5 × 10 ± 3.0 colonies/10^7^ plating cells versus 1.4 × 10^2^ ± 1.4 × 10 colonies/10^7^ plating cells for Δ*ssb* and DP-1, respectively). Previously, we showed that *S. acidocaldairus* Δ*ssb* exhibited a cold-sensitive growth phenotype, indicating that SSB function for cellular growth and production was more important at low growth temperatures than at high growth temperatures [37]. Therefore, we speculated that if the SSB function was required for mutation avoidance at low growth temperatures, the mutation rate would increase when the cells were precultivated at low temperatures. Notably, when cells were used that had been precultivated at 60 °C, a temperature that had only limited effects on the growth of Δ*ssb* compared to that of the parent strain DP-1 [37], the Δ*ssb* mutation rate was 29-fold higher than that of DP-1 (3.5 × 10^2^ ± 1.1 × 10^2^ colonies/10^7^ cells versus 1.2 × 10 ± 4 colonies/10^7^ cells for Δ*ssb* and DP-1, respectively). Thus, these results indicate that the loss of the SSB function causes a loss in genetic accuracy in *S. acidocaldarius* at low growth temperatures.

### 2.3. SSB Is Involved in Reliable HR Processivity in S. acidocaldarius

**Figure 1:**
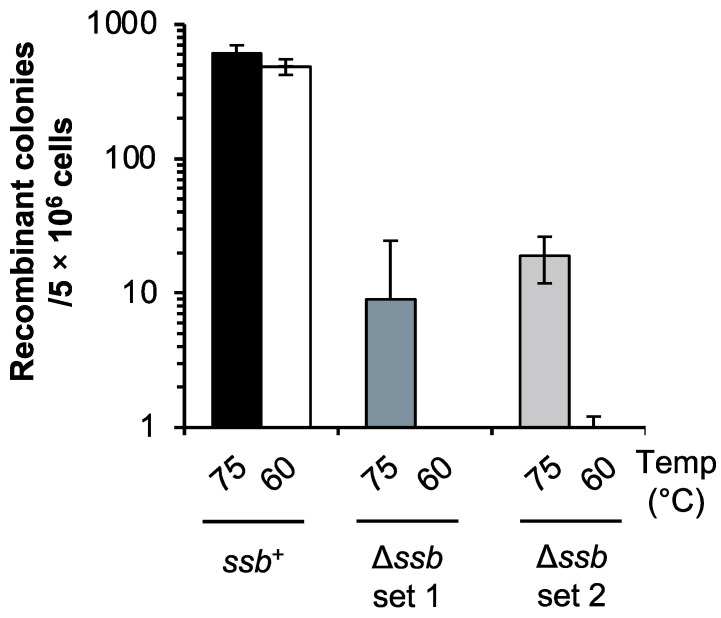
HR frequency in the *ssb*-deleted strain through the mating test. Recombinant colonies resulted from mating of uracil-auxotrophic strains. Recombination between two strains successfully restored the uracil-proficient phenotype. Numbers 75 and 60 indicate the cultivation temperature (°C) before mating. Mating was performed as follows: *Ssb*^+^, DP-1 and DP-2. Δ*ssb* set 1; DP-5 and DP-11-1. Δ*ssb* set 2; DP-5 and DP-11-3. Error bars represent ±SD calculated using three biological replicates.

We examined whether SSB was important for HR in vivo through a mating test. The mating test was performed according to the experiment Grogan [44] (Figure 1). When each of the uracil-auxotrophic parent strains (*ssb*^+^), DP-1 and DP-2 were cultivated at 75 °C, they mated, and 6.1 × 10^2^ ± 9.1 × 10 recombinant colonies grew (Figure 1). In the case of Δ*ssb*, remarkably, 9 ± 1.6 × 10 and 1.9 × 10 ± 7 recombinant colonies appeared (Figure 1: Δ*ssb* set1; DP-5 and DP-11-1. Δ*ssb* set2; DP-5 and DP-11-3). When each uracil-auxotrophic strain of Δ*ssb*, cultivated at 60 °C, was mated, notably, the recombinant colonies hardly grew (Figure 1). In contrast, the number of recombinant colonies of the *ssb*^+^ parent strain cultivated at 60 °C was the same as that cultivated at 75 °C (4.9 × 10^2^ ± 6.4 × 10) (Figure 1). These results suggest that SSB was involved in DNA transfer and/or HR in *S. acidocaldarius* and its function were especially essential at a low temperature.

To obtain direct evidence of the involvement of SSB in HR in vivo, we also examined HR frequency via the double-crossover of HR using a linear marker cassette (pyrElacS800) in Δ*ssb*. In this test, the selectable marker (*lacS*-*pyrE*) could only be maintained if it was integrated into the host genome by HR via double crossover between the linear marker cassette and the chromosome at the 5′ and 3′ homologous regions of the target locus (see Materials and Methods section) [38]. The autonomously replicating vector pSAV2 containing *pyrE* was used as a control to determine the transformation efficiency. As a control, the apparent difference in the transformation efficiency of Δ*ssb* and DP-1 was not observed (1.4 × 10^4^ and 2.4 × 10^4^ transformants/1 μg of pSAV2 for Δ*ssb* and DP-1, respectively) (n = 2), suggesting that the DNA uptake capacity via the electroporation of both strains was similar. In contrast, the HR frequency of Δ*ssb* was 5.6-fold lower than that of DP-1 (4.2 × 10 ± 4.1 × 10 and 2.3 × 10^2^ ± 1.7 × 10^2^ transformants/1 μg of pyrElacS800 for Δ*ssb* and DP-1, respectively) (n = 8) (Figure S2). These results suggest that SSB was involved in the HR process in *S. acidocaldarius* in vivo, which is consistent with the results of the mating test (Figure 1). For this reason, we considered that the decrease in recombinant colonies of Δ*ssb* in the mating test implied the involvement of SSB in HR but not in DNA transfer.

### 2.4. Sensitivity of Gene-Deleted Strains to UV-B Irradiation

**Figure 2:**
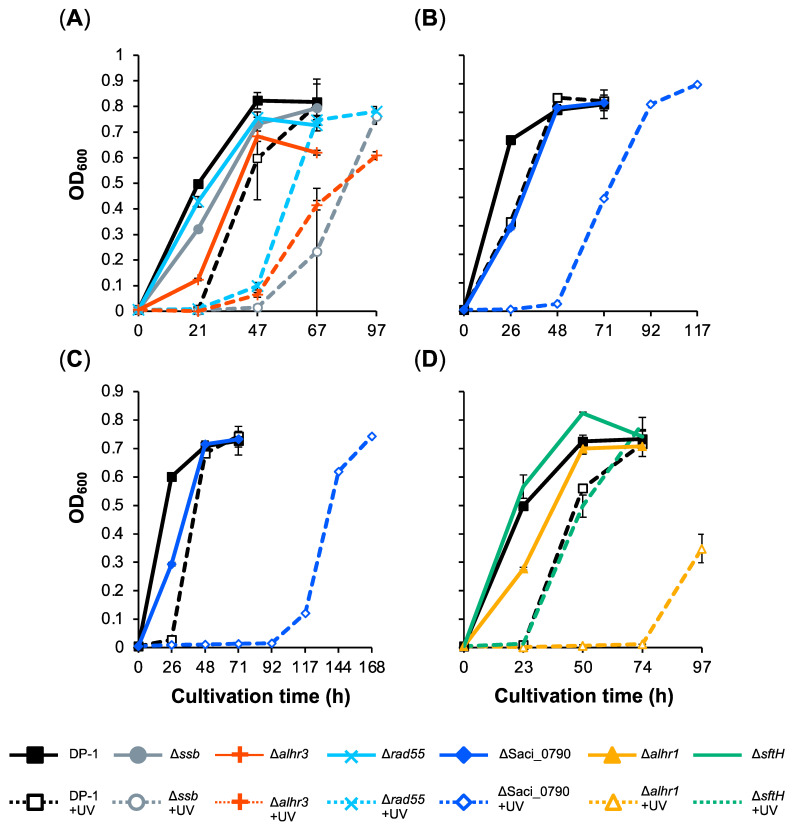
Growth curve of gene-deletion strains after UV-B irradiation. Each overnight culture of DP-1 (parent strain), Δ*ssb*, Δ*alhr1,* Δ*alhr3*, Δ*sftH*, Δ*rad55*, and ΔSaci_0790 was irradiated with UV-B light (800 (**B**) and 1200 J/m^2^ (**A**,**C**,**D**); +UV represents a UV-treated sample) followed by inoculation in a liquid medium and cultivation at 75 °C with shaking. Growth curves for Δ*ssb*, Δ*alhr3*, and Δ*rad55* are indicated in (**A**), those of ΔSaci_0790 are shown in (**B**,**C**), and those of Δ*alhr1* and Δ*sftH* are displayed in (**D**). Error bars represent ±SD calculated using three biological replicates.

Helix-distorting DNA lesions (such as photoproducts, intrastrand crosslinks, and bulky adducts) are induced by UV irradiation and DNA-damaging agents. In addition to studying SSB, we investigated the involvement of the helicase *Sac*aLhr1; the putative helicases aLhr3 and SftH; the recombinase mediator Rad55; and the hypothetical protein Saci_0790 as putative SSB-interacting proteins in the repair of helix-distorting DNA lesions. The DNA photolyase-deficient strain DP-1 and its derivatives did not exhibit photoreactivation under light conditions [43]. To characterize the UV sensitivity of the gene-deleted strains Δ*ssb*, Δ*alhr1*, Δ*alhr3*, Δ*sftH*, Δ*rad55*, and ΔSaci_0790, we investigated their growth properties, in parallel to those of the parental strain DP-1, in a liquid medium under three different levels of UV-B irradiation (zero, 800 and 1200 J/m^2^) (Figure 2). In this batch, the growth of Δ*ssb*, Δ*alhr1*, Δ*alhr3*, and ΔSaci_0790 using mock-treated samples was slightly retarded (Figure 2). After UV irradiation at 1200 J/m^2^ (Figure 2A), the growth retardation of Δ*ssb*, Δ*alhr3*, and Δ*rad55* was observed. Similarly, the growth retardation of ΔSaci_0790 was observed after UV irradiation at 800 J/m^2^ (Figure 2B). Notably, when ΔSaci_0790 and Δ*alhr1* were exposed to UV irradiation at 1200 J/m^2^, growth was significantly delayed (Figure 2C,D). No marked difference was observed between the growth of Δ*sftH* and DP-1 after UV irradiation at 1200 J/m^2^ (Figure 2D). These results indicate that Δ*ssb*, Δ*alhr3*, and Δ*rad55* are sensitive to UV-B irradiation, that Δ*alhr1* is more sensitive, and that ΔSaci_0790 is markedly sensitive.

**Figure 3:**
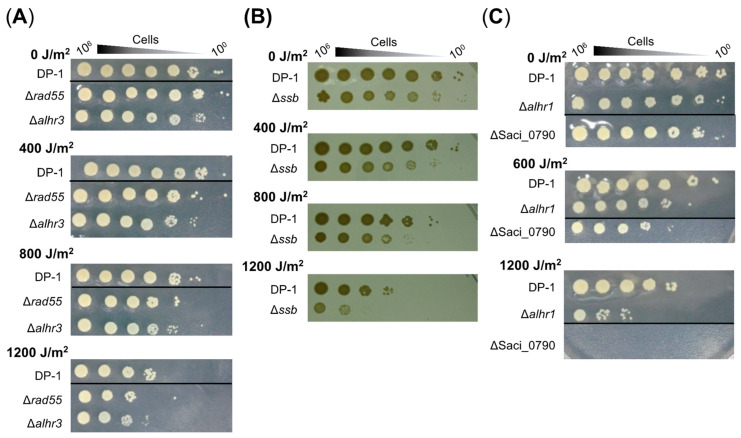
UV survival in the knockout strains. Each overnight culture of DP-1 (parent strain), Δ*rad55*, Δ*alhr3*, Δ*ssb*, Δ*alhr1*, and ΔSaci_0790 was exposed to UV-B light (0, 400, 600, 800, and 1200 J/m^2^), and aliquots were serially diluted (10^0^–10^−6^ corresponding to 10^6^–10^0^ cells) and spotted onto plates. Plates were incubated at 75 °C. UV survival for Δ*rad55* and Δ*alhr3*, Δ*ssb*, and Δ*alhr1* and ΔSaci_0790 is shown in (**A**–**C**), respectively. Experiments were repeated three times with similar results.

The UV-B survival of gene-deleted strains was also examined by means of a spotting test after UV irradiation (400–1200 J/m^2^), and the results are presented in Figure 3. The mock-treated strains are indicated in the figure (0 J/m^2^). Compared with DP-1, Δ*rad55* and Δ*alhr3* exhibited marginal sensitivity to UV-B irradiation (400–1200 J/m^2^) (Figure 3A). Although the colony number for Δ*ssb* was nearly the same as that of DP-1 after UV irradiation (400–1200 J/m^2^) (Figure 3B), at 1200 J/m^2^, the colony size for Δ*ssb* was significantly smaller than that of DP-1 and that of mock-treated Δ*ssb*. In contrast, compared with DP-1, Δ*alhr1* survived with low colony numbers after UV irradiation (600 and 1200 J/m^2^) (Figure 3C). Similar to Δ*alhr1*, ΔSaci_0790 also exhibited sensitivity to UV irradiation at 600 J/m^2^ (Figure 3C). Notably, colonies of ΔSaci_0790 hardly grew after UV irradiation at 1200 J/m^2^ (Figure 3C). No difference in survival under UV irradiation was observed between Δ*sftH* and DP-1 (1200 J/m^2^) (Figure S3). These results reveal that Δ*rad55* and Δ*alhr3* exhibit slight sensitivity to UV-B irradiation, that Δ*ssb* and Δ*alhr1* are sensitive, and that ΔSaci_0790 is markedly sensitive. For this reason, the UV sensitivity of Δ*rad55*, Δ*alhr3*, Δ*ssb*, Δ*alhr1*, and ΔSaci_0790 were supported by not only the results of the growth curve for gene-deleted strains after UV irradiation but also the results of the spotting test after UV irradiation (Figure 2 and Figure 3).

### 2.5. SSB and SacaLhr1 May Be Involved in the Removal of UV-Induced DNA Photoproducts

**Figure 4:**
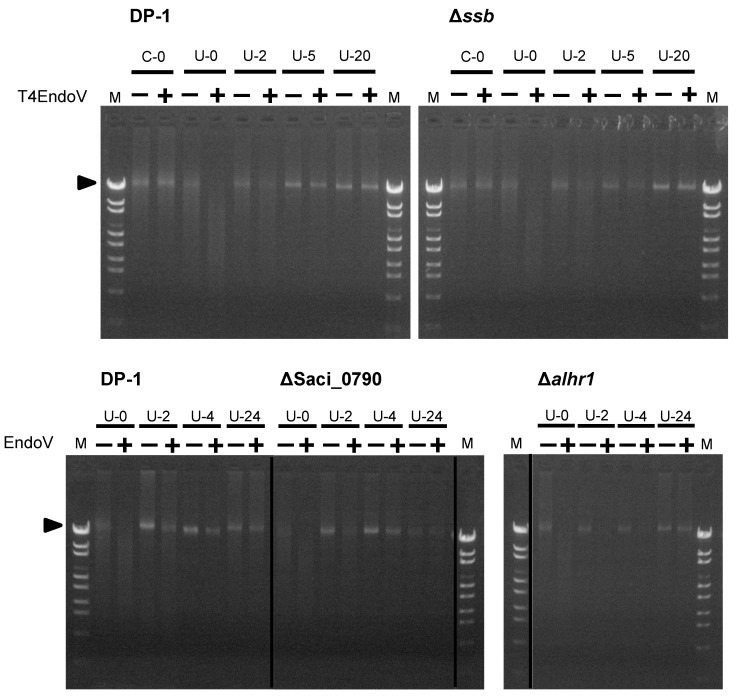
Analysis of the DNA repair capacity of cyclobutane pyrimidine dimers (CPDs) in Δ*ssb*, Δ*alhr1*, and ΔSaci_0790. The genomic DNA isolated from mock-treated (C-0) and irradiated cultures of each strain at each time point (U-0–24, where numbers mean cultivation time [hours] after UV irradiation [1200 J/m^2^]) was cut with T4 EndoV (lane +) or mock-treated (lane −). The genomic DNA was denatured followed by loading on a 1% agarose gel stained with ethidium bromide. The arrow indicates the position of the bands containing uncut genomic DNA.

Because we observed the sensitivity of Δ*ssb*, Δ*alhr1*, and ΔSaci_0790 to UV-B irradiation (Figure 2 and Figure 3), the repair capacities of these strains in regard to cyclobutane pyrimidine dimers (CPDs), as UV-induced DNA photoproducts, were characterized according to the experiment of Suzuki and Kurosawa [45]. This was achieved through a specific digestion assay for CPD-containing DNA. The parent strain DP-1 and gene-deleted strains Δ*ssb*, Δ*alhr1*, and ΔSaci_0790 were irradiated with UV-B light (1200 J/m^2^) before being immediately incubated at 75 °C. Genomic DNA was extracted from the cultures at various time points after UV irradiation and was treated with T4 EndoV: an endonuclease that specifically introduces a nick at the CPD site. The denatured genomic DNA was subsequently monitored using agarose gel electrophoresis (Figure 4). For example, genomic DNA, which was isolated from the mock-treated samples of DP-1 and Δ*ssb* was not digested (lane C-0+), while genomic bands in the genomic DNA, isolated from the irradiated cultures, disappeared (lane U-0+). When the cultures were incubated at 75 °C for 2 h after UV irradiation, part of the DP-1 and Δ*ssb* genomic DNA appeared at the position of the uncut genomic DNA (arrow, lane U-2+), indicating that the repair of the CPDs had already started. Most of the CPDs were removed from the DP-1 genomic DNA within 5 h (lane U-5). In contrast, the number of CPDs in the Δ*ssb* genomic DNA after 5 h of cultivation seemed to remain nearly the same as the quantity had been after 2 h (lanes U-2 and U-5). After cultivation for 20 h (lane U-20), most of the DP-1 and Δ*ssb* DNA, was not digested. In the case of Δ*alhr1* and ΔSaci_0790, although the ΔSaci_0790 CPD repair capacity was the same as that of DP-1 (lanes U-2–4), that of Δ*alhr1* seemed to be relatively low (lanes U-2–4). However, both Δ*alhr1* and ΔSaci_0790 were able to repair most of the CPDs (U-24). The results suggest that SSB and *Sac*aLhr1 were involved in *S. acidocaldarius* CPD repair but also indicates that the individual functions of SSB, *Sac*aLhr1 and Saci_0790 are not essential for the removal of CPDs.

If SSB is directly involved in the repair of CPDs but not in another cellular process, it appears that the transformation efficiency of the *ssb*-deleted strain decreases in comparison with that of the parent strain, even if only plasmid DNA is exposed to UV irradiation. For this reason, we estimated the transformation efficiency (the number of transformants per 1 μg DNA) using UV-B-irradiated pSAV2 (3600 J/m^2^) or mock-treated pSAV2. DP-1 or Δ*ssb* was transformed with 50 ng of plasmid DNA by electroporation (15 kV/cm, 9 ms) and spread on XT selective plates. The transformation efficiency was calculated by counting colonies that appeared. The transformation efficiency of Δ*ssb* when using UV-irradiated pSAV2 was approximately 7.8-fold lower than that of mock-treated pSAV2 (1.8 × 10^3^ colonies/1 μg of UV-irradiated pSAV2 and 1.4 × 10^4^ colonies/1 μg of pSAV2) (n = 2), whereas that of DP-1 was nearly unchanged (1.7 × 10^4^ colonies/1 μg of UV-irradiated pSAV2 and 2.4 × 10^4^ colonies/1 μg of pSAV2) (n = 2). This result is consistent with the result that was produced by the decreasing CPD repair capacity in Δ*ssb* (Figure 4).

### 2.6. Sensitivity of Gene-Deleted Strains to Helix-Distorting DNA Lesions

**Figure 5:**
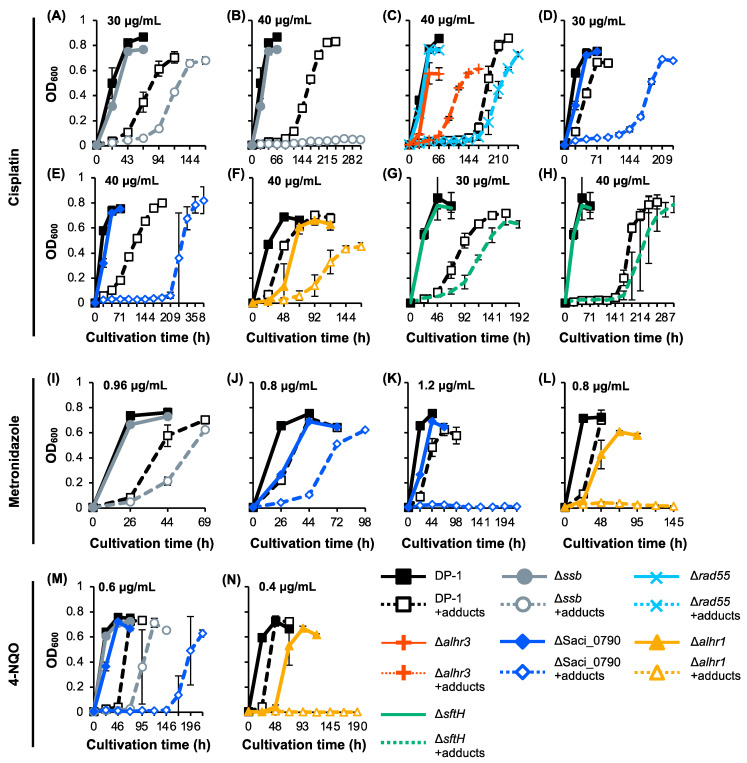
Growth curve of the knockout strains in the presence of DNA adducts. Each overnight culture of DP-1 (parent strain), Δ*ssb*, Δ*rad55*, Δ*alhr3*, ΔSaci_0790, Δ*alhr1*, and Δ*sftH* was inoculated in liquid medium in the presence of cisplatin (**A**–**H**) (30 (**A**,**D**,**G**) and 40 μg/mL (**B**,**C**,**E**,**F**,**H**)) metronidazole at (**I**–**L**) 0.8 (**J**,**L**), 0.96 (**I**), and 1.2 mg/mL (**K**)), and 4-NQO (**M**,**N**) (0.4 (**N**) and 0.6 μg/mL (**M**)), respectively, before being cultivated at 75 °C with shaking. Growth curves of Δ*ssb* (**A**,**B**,**I**,**M**), Δ*rad55* and Δ*alhr3* (**C**), ΔSaci_0790 (**D**,**E**,**J**,**K**,**M**), Δ*alhr1* (**F**,**L**,**N**), and Δ*sftH* (**G**,**H**) are shown. Solid and dotted (+adducts) lines indicate growth curves in the absence or presence of DNA adducts, respectively. Error bars represent ±SD calculated using three biological replicates.

To investigate whether SSB, *Sac*aLhr1, aLhr3, SftH, Rad55, and Saci_0790 were involved in the repair of other types of helix-distorting DNA lesions (intra-strand crosslink [cisplatin] and bulky adducts [metronidazole and 4-nitroquinoline N-oxide 4-NQO]), the growth properties of the gene-deleted strains were characterized in the presence or absence of helix-distorting DNA-damaging agents and were compared with those of the parent strain DP-1 (Figure 5). In the absence of DNA-damaging agents in this batch, we observed that the growth of Δ*alhr3* and ΔSaci_0790 was delayed, that the final cell density of Δ*alhr3* was relatively low (Figure 5C–E,J,K,M), and that the growth of Δ*alhr1* was significantly retarded (Figure 5F,L,N). The cisplatin sensitivity test showed that the growth of DP-1 was inhibited at 30 μg/mL cisplatin (Figure 5A,D,G). The growth of Δ*ssb* and Δ*sftH* was delayed for more than that of DP-1 (Figure 5A,G), and that of ΔSaci_0790 was markedly delayed (Figure 5D). In the presence of 40 μg/mL cisplatin, there was more of a delay in the growth of Δ*rad55*, Δ*alhr1*, and Δ*sftH* than in that of DP-1 (Figure 5C,F,H), and the final cell density of Δ*alhr1* was lower than that of DP-1 (Figure 5F). A marked growth retardation was also detected for ΔSaci_0790 (Figure 5E). Notably, Δ*ssb* did not grow (Figure 5B). In contrast, Δ*alhr3* exhibited tolerance to cisplatin, and the growth was observed to be earlier than that of DP-1 (Figure 5C). The results indicate that Δ*alhr1*, Δ*sftH*, and Δ*rad55* were sensitive to cisplatin and that Δ*ssb* and ΔSaci_0790 were markedly sensitive.

The metronidazole sensitivity test at 0.8–1.2 μg/mL revealed that growth retardation for Δ*ssb* and for ΔSaci_0790, compared to DP-1, was observed in the presence of 0.96 and 0.8 mg/mL metronidazole, respectively (Figure 5I,J). Notably, there was no growth observed for ΔSaci_0790 and Δ*alhr1* in the presence of 1.2 and 0.8 mg/mL metronidazole, respectively (Figure 5K,L). No clear difference was observed between the growth of Δ*alhr3*, Δ*sftH*, and Δ*rad55* and the growth of DP-1 in the presence of 0.32–0.96 mg/mL metronidazole (Figure S4). These results demonstrate that Δ*ssb* was sensitive to metronidazole and that Δ*alhr1* and ΔSaci_0790 were markedly sensitive.

The 4-NQO sensitivity test at 0.3–0.6 μg/mL revealed growth retardation in Δ*ssb* and significant growth retardation in ΔSaci_0790 compared with the growth of DP-1 in the presence of 0.6 μg/mL 4-NQO (Figure 5M). Remarkably, at even 0.4 μg/mL 4-NQO, Δ*alhr1* exhibited no growth (Figure 5N). No clear difference was observed between the growth of Δ*alhr3*, Δ*sftH*, and Δ*rad55* and that of DP-1 in the presence of 0.3–0.5 μg/mL 4-NQO (Figure S4). These results reveal that Δ*ssb*, ΔSaci_0790, and Δ*alhr1* were, respectively, sensitive, more sensitive, and markedly sensitive to 4-NQO.

### 2.7. H_2_O_2_ Survival of Gene-Deleted Strains

**Figure 6:**
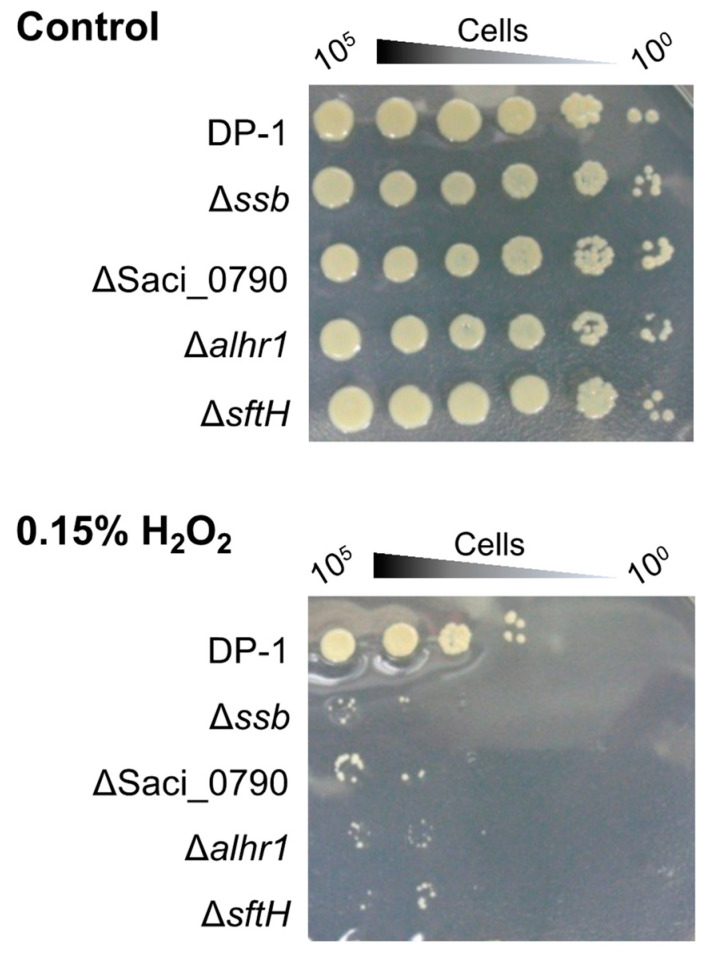
Hydrogen peroxide survival of gene-deleted strains. After each of the cultures was treated with hydrogen peroxide (0.15%) followed by the preparation of diluted samples (10^−1^–10^−5^ corresponding to 10^5^–10^0^ cells), the samples were spotted on plates and cultivated at 75 °C. Controls indicate mock-treated samples. Experiments were repeated three times with similar results.

To examine the sensitivity of Δ*ssb*, Δ*alhr1*, Δ*alhr3*, Δ*sftH*, Δ*rad55*, and ΔSaci_0790 to H_2_O_2,_ which produces the hydroxyl radical that has the potential to induce double-strand breaks (DSB) and oxidative stress [46], mock- and H_2_O_2_-treated (zero and 0.15%) cells were spotted on plates (Figure 6). An H_2_O_2_ survival test revealed that colonies of Δ*ssb*, ΔSaci_0790, Δ*alhr1*, and Δ*sftH* hardly grew in comparison with DP-1 colonies (Figure 6). In contrast, the sensitivity of Δ*alhr3* and Δ*rad55* to H_2_O_2_ was the same as that of DP-1. The results indicate that Δ*ssb*, Δ*alhr1*, Δ*sftH*, and ΔSaci_0790 were markedly sensitive to H_2_O_2_.

### 2.8. SacaLhr1 and Saci_0790 Are Required for Robust Growth at High and Low Growth Temperatures, Respectively

**Figure 7:**
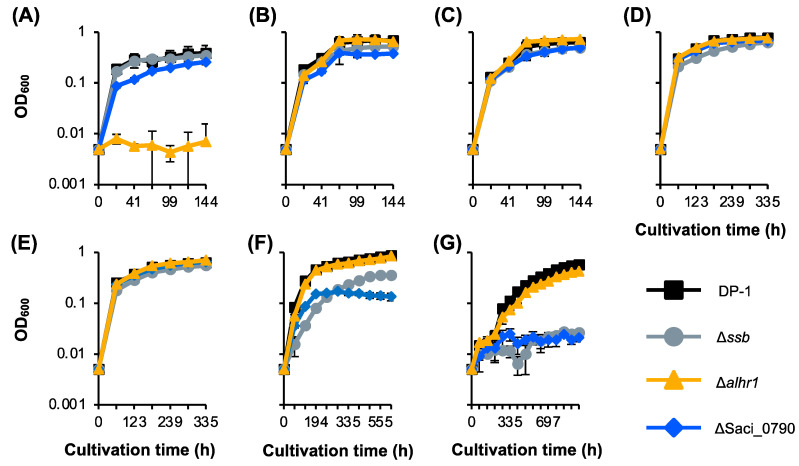
Growth curve of gene-deletion strains at various temperatures. Cell density as a function of cultivation time is shown for various temperatures (**A**–**G**): (**A**) 80 °C. (**B**) 75 °C. (**C**) 70 °C. (**D**) 65 °C. (**E**) 60 °C. (**F**) 55 °C. (**G**) 50 °C. Error bars represent ±SD calculated using three biological replicates. The data for DP-1 and Δ*ssb* are the same as those of [38].

Previously, we reported that Δ*ssb* exhibited cold sensitivity, i.e., an increase in the minimal growth temperature [38]. The growth of Δ*alhr1* and ΔSaci_0790, in parallel with that of Δ*ssb*, in the liquid medium was compared to that of DP-1 over a wide temperature range (50–80 °C) (Figure 7A–G) [38]. Because Δ*alhr1* and ΔSaci_0790 exhibited sensitivity to a wide variety of DNA damage types (Figure 2, Figure 3, Figure 5 and Figure 6), we focused on these two strains. The growth curves for Δ*alhr1* and ΔSaci_0790 were indistinguishable from that of DP-1 over a wide temperature range (75–60 °C) (Figure 7B–E). At 80 °C, the growth of ΔSaci_0790 was slightly slower than that of DP-1, and Δ*alhr1* hardly grew (Figure 7A). In the case of low growth temperatures (below 55 °C), Δ*alhr1* grew normally compared with DP-1 (Figure 7F,G). Notably, at 55 °C, ΔSaci_0790 grew much slower than the parent strain, and at 50 °C, ΔSaci_0790 could not grow (Figure 7F,G). The growth defect in ΔSaci_0790 at a low temperature was similar to the case of Δ*ssb* (Figure 7F,G). The results indicate that Δ*alhr1* and ΔSaci_0790 exhibited hot and cold sensitivities, respectively, i.e., a decrease and an increase in the maximal and minimal growth temperatures, respectively.

Similarly, we previously reported that Δ*ssb* exhibited heat-shock sensitivity, i.e., a decrease in heat-shock survival [38]. Therefore, we investigated the sensitivity of Δ*alhr1* and ΔSaci_0790, in parallel with DP-1 and Δ*ssb*, to heat-shock treatment (Figure S5). When DP-1, Δ*alhr1*, and ΔSaci_0790 were treated with heat shock at 90 °C for 3 min, most of the cells survived (Figure S5). In this condition, compared to the DP-1 colonies, few Δ*ssb* colonies grew after heat-shock treatment (Figure S5). We concluded that Δ*alhr1* and ΔSaci_0790 did not exhibit sensitivity to heat shock.

### 2.9. Cultivation Temperature Markedly Affects the Susceptibility of the ssb-Deleted Strain to DNA Damage

On the basis of the mutation frequency and mating test results for Δ*ssb* (Figure 1), we investigated whether cultivation temperature affected Δ*ssb*’s susceptibility to DNA damage. In this test, we defined the pre-cultivation temperature as the cultivation temperature when inoculums were prepared and the post-cultivation temperature as the cultivation temperature after inoculation, following UV irradiation or exposure to DNA adducts (Figures S3 and S4).

The UV sensitivity test indicated that at a lower UV dose (600 J/m^2^) (Figure S6A–D), the post-cultivation temperature did not affect the UV sensitivity of Δ*ssb* when the pre-cultivation temperature was 75 °C (Figure S6A,B). In contrast, when the pre-cultivation temperature was 60 °C (Figure S6C,D), notably, the growth of Δ*ssb* was significantly delayed after UV irradiation. This tendency was accelerated by decreasing the post-cultivation temperature (from 75 °C to 60 °C). Under these conditions, the growth of the *ssb*+ parent was not delayed (Figure S6C,D). At a higher UV dose (1200 J/m^2^) (Figure S6E–H), remarkably, no growth was observed in Δ*ssb* in this experiment (Figure S6F–H) except for when the pre- and post-cultivation temperatures were both 75 °C (Figure S6E). In addition, the UV sensitivity of DP-1 seemed to also increase under lower pre- and post-cultivation temperatures (Figure S6E–H). The results demonstrate that both pre- and post-cultivation temperatures affect the UV sensitivity of Δ*ssb* especially and that, compared with the pre-cultivation temperature, the post-cultivation temperature markedly modulates Δ*ssb* sensitivity (Figure S6E,F).

Next, we focused on the effect of the post-cultivation temperature of Δ*ssb* on sensitivity to DNA adducts (Figure S7). Similarly, we investigated the growth properties of Δ*ssb* in the liquid medium in the presence of DNA adducts at both 60 °C and 75 °C (Figure S7). In this test, the pre-cultivation temperature was 75 °C. Notably, a marked Δ*ssb* growth retardation was observed in the presence of DNA adducts [cisplatin (30 and 40 μg/mL), metronidazole (1.2 mg/mL), and 4-NQNO (0.5 μg/mL)] at 60 °C but not at 75 °C (Figure S7). These results indicate that Δ*ssb* sensitivity to DNA adducts was markedly increased at low growth temperatures.

## 3. Discussion

The study of DNA repair in hyperthermophiles has the potential to identify novel and unique proteins that are involved in genetic information maintenance systems. Recent genetic studies [6,15,16,17] have raised two questions: (i) How does *S. acidocaldarius*, whose EndoMS/NucS is not an essential component for mutation avoidance, consistently maintain genome stability? (ii) How does HA repair helix-distorting DNA lesions that are generally repaired by the NER pathway in other organisms? To address these questions, we focused on SSB and the putative SSB-interacting proteins *Sac*aLhr1, aLhr3, SftH, Rad55, and Saci_0790 as relevant candidates for genetic characterization in *S. acidocaldarius*.

EndoMS was identified as an endonuclease cleaving dsDNA containing mismatched bases, suggesting that EndoMS is involved in mismatch repair in HA [22,47,48]. Genetic analysis by Ahmad et al. [23] demonstrated that EndoMS was responsible for mutation avoidance in *Sa*. *islandicus*. In contrast, our past genetic work indicated that EndoMS was not implicated in mutation avoidance in *S. acidocaldarius,* which also belongs to the *Sulfolobales* order [17]. The high mutation rate in the Δ*ssb* cells in the present study indicates that SSB is actually necessary for genetic accuracy in *S. acidocaldarius*. As another protein involved in mutation avoidance, a recent genetic study by Miyabayashi et al. [49] revealed that DNA polymerase B1-binding protein 1 is important for mutation avoidance in *S. acidocaldarius*. For this reason, our present study is the third report of a protein being involved in mutation avoidance in HA. Interestingly, Δ*ssb* exhibited a high mutation rate when the cells were pre-cultivated at a lower growth temperature (60 °C) but not at a higher growth temperature (75 °C). However, it is unclear why a loss in the SSB function caused a high mutation rate. At this stage, we suppose that (i) the destabilization of the dsDNA region as the SSB function may be essential at lower temperatures for destabilizing the dsDNA region that contains mismatched bases, and the loss of this SSB function as a first step of mutation avoidance may cause genome instability; (ii) this SSB function is partially complemented by thermal denaturation at high temperatures, resulting in Δ*ssb* maintaining genome stability at high temperatures. We did not investigate what type of mutations were dominant in Δ*ssb*, and further analysis based on sequencing is needed to elucidate the repair process. Regarding other candidates for mutation avoidance in HA, Bell and Grogan [50] isolated *S. acidocaldarius* mutant strains that exhibited abnormally high rates of spontaneous mutation but not sensitivity to DNA-damaging agents, suggesting that other unknown proteins that are involved in mutation avoidance exist in *S. acidocaldarius*. It seems that the genome sequencing of candidate strains followed by genetic and biochemical studies could be important for the identification of several key proteins for mutation avoidance in HA.

To date, it remains unclear whether SSB is involved in HR in archaea because there is no direct evidence of its involvement in HR in vivo. In our genetic assay, using the selectable marker, we noted that Δ*ssb* exhibited a defect in HR frequency (5.6-fold decrease) when compared to the parental strain (Figure S2). A similar decrease in HR frequency was demonstrated by the results of a mating test (Figure 1) and the deletion of the gene encoding *Sac*aLhr1 in this archaeon [38]. In our genetic assay in the present study, the integration of the selectable marker *lacS*-*pyrE* in the genomic locus by HR via a double crossover may be composed of the end resection of the linear marker cassette, strand exchange, the formation of double HJs, branch migration, and HJ resolution. Thus, the decrease in HR frequency through the deletion of the *ssb* gene suggests that SSB is directly involved in the HR process in vivo. Notably, when the Δ*ssb* cells were precultured at a lower temperature and mated, the HR capacity in Δ*ssb* was completely abolished (Figure 1), suggesting that SSB plays an essential role in HR in vivo. However, at this stage, it is not clear why the cellular conditions in Δ*ssb* at lower temperatures completely inhibit the HR process.

The sensitivities of the gene-deleted strains to DNA damage are summarized in Table 1. In addition to the role of SSB in mutation avoidance and HR in vivo, our phenotypic characterization of Δ*ssb* against DNA damage demonstrated sensitivities to a wide variety of helix-distorting DNA lesions, including UV-induced DNA damage, intrastrand crosslinking, and bulky adducts (Table 1, Figure 2A, Figure 3B and Figure 5A,B,I,M), indicating that SSB is involved in the repair of helix-distorting DNA lesions. We suppose that the loss of the SSB function caused a partial deficiency in HR-mediated stalled-fork DNA repair because SSB was involved in the HR process, resulting in its broad sensitivity. The sensitivity of Δ*ssb* to helix-distorting DNA damaging agents and the decreasing capacity to repair CPDs (Table 1, Figure 2A, Figure 3B, Figure 4 and Figure 5A,B,I,M) do not directly support the hypothesis that SSB acts as a dsDNA containing helix-distorting DNA lesion melting proteins at the first step in an unknown NER process in *S. acidocaldarius*. At the very least, our genetic study and a previous in vitro study [36] indirectly support the hypothesis and do not refute it. We suppose that SSB participates in both HR and unknown NER processes for the repair of helix-distorting DNA lesions. At this stage, XPF and NucS also have potential as other candidates for unknown NER processes [17]. In addition, Δ*ssb* exhibited significant sensitivity to H_2_O_2_, which has the potential to induce DSB and oxidative stress (Table 1 and Figure 6), and this result is consistent with the loss of HR function because it is generally known that the HR function is required for DSB repair.

We discussed the reason why a lower post-cultivation temperature caused marked Δ*ssb* susceptibility to helix-distorting DNA damaging agents (Figures S3 and S4). We supposed that the role of the “destabilization of dsDNA” SSB function and thermal denaturation at a high temperature might be complementary. For this reason, it seemed plausible that thermal denaturation at high temperatures but not at lower temperatures partially compensated for the loss of SSB function in DNA repair (Figures S6 and S7). In conclusion, SSB is significantly important for the repair of helix-distorting DNA lesions at lower temperatures.

In addition to the important role of SSB in DNA repair, this study identified two novel key proteins, namely, *Sac*aLhr1 and hypothetical Saci_0790, as being involved in the repair of helix-distorting DNA lesions in *S. acidocaldarius* (Table 1). In contrast to *Sac*aLhr1, whose homologs are nearly ubiquitously distributed in archaea [40], Saci_0790 homologs may have a limited distribution among members of the *Sulfolobales*, *Acidilobales*, and *Desulfurococcales* orders. The cold-sensitive growth phenotype and heat-shock sensitivity of Δ*ssb* have been previously reported [37], and it seems that the functions of *Sac*aLhr1 and Saci_0790 are not required for survival against transient heat-shock stress (Figure S5). Interestingly, ΔSaci_0790 exhibits the same cold-sensitive growth phenotype (Figure 7). In contrast, Δ*alhr1* exhibits a growth defect at 80 °C (Figure 7). Δ*alhr1* and ΔSaci_0790 exhibit sensitivities to a wide variety of helix-distorting DNA lesions (Table 1), suggesting that *Sac*aLhr1 and Saci_0790 may be involved in DNA repair for the maintenance of genome integrity at higher and lower growth temperatures, respectively. In addition, similar to Δ*ssb*, we observed that Δ*alhr1* and ΔSaci_0790 were significantly sensitive to H_2_O_2_, suggesting that *Sac*aLhr1 and Saci_0790 are involved in DSB repair and/or the response to oxidative stress. The results for *Sac*aLhr1 may be consistent with the results for the sensitivity of ΔSiRe_1605 (*Sac*aLhr1 homolog) in *Sa*. *islandicus* to an alkylating agent methyl methanesulfonate (MMS) [52] because the MMS treatment of *Saccharolobus* cells induces DNA fragmentation [53]. Given that *Sac*aLhr1 may be involved in HR in vivo [38], we hypothesized that Δ*alhr1* exhibited sensitivity to H_2_O_2_ due to the partial deficiency of the HR function.

Unlike Δ*ssb*, Δ*alhr1*, and ΔSaci_0790, which exhibit a wide variety of sensitivities to helix-distorting DNA damaging agents, Δ*alhr3*, Δ*sftH*, and Δ*rad55* are slightly sensitive only to UV irradiation and/or cisplatin (Table 1, Figure 2A, Figure 3A and Figure 5C,G,H). The most obvious Δ*sftH* phenotype involved a significant sensitivity to H_2_O_2_ (Table 1 and Figure 6), suggesting that SftH was involved in DSB repair and/or response to oxidative stress.

As this study focused on genetic characterization, we did not biochemically analyze the interaction between SSB and putative SSB-interacting proteins. Given the previous phylogenetic analysis of SF2 helicases [39] and aLhrs [40], aLhr1, aLhr2, aLhr3, and SftH seemed to have evolved from a common ancestral helicase. Interestingly, aLhr1, aLhr3, and SftH, but not aLhr2, were previously copurified with the ssDNA–SSB complex in a pull-down experiment [36]. To understand when the interaction between SSB and these helicases developed and when the function of this interaction in genome integrity diversified during evolution, it is important to characterize whether aLhr1, aLhr2, aLhr3, and SftH actually interact with SSB.

A study of DNA repair in HA is important for understanding how life maintains genetic information under extreme environments. Compared with the DNA repair pathways of other organisms, HR-mediated DNA repair seems very important for genome integrity in HA [6]. The present study demonstrated that SSB was involved in mutation avoidance, HR, and the repair of a wide variety of helix-distorting DNA lesions in the thermophilic crenarchaeon *S. acidocaldarius*. Additionally, a novel helicase *Sac*aLhr1, which participates in HR in vivo [38], and the hypothetical protein Saci_0790 were shown to also be very important for the repair of helix-distorting DNA lesions in *S. acidocaldarius*. Thus, this study provides insight into novel key proteins in mutation avoidance and into the repair of helix-distorting DNA lesions in HA. Furthermore, this study is required to understand how SSB, *Sac*aLhr1, and Saci_0790 are involved in the maintenance of genome integrity in this archaeon.

## 4. Materials and Methods

### 4.1. Strains and Growth Conditions

The strains used in this study are listed in Table 2. The growth conditions were previously reported [43]. The *S. acidocaldarius* pyrimidine-auxotrophic strain DP-1 lacking the restriction endonuclease *Sua*I- and DNA photolyase Phr-encoding gene (Δ*pyrE* Δ*suaI* Δ*phr*) was used as the parent strain in this study [43]. The DP-1 strain and its derivative gene-deleted strains were cultivated in 6 mL of an XTU medium (a xylose and tryptone [XT] medium [46] supplemented with 0.02 g/L uracil) (pH 3) at 75 °C with or without shaking at 160 rpm. The XTU medium supplemented with 50 μg/mL 5-FOA (XTUF) was used for counterselection with the pop-out recombination method [43,54].

### 4.2. General DNA Manipulation

The reagents used in these experiments were prepared as previously described [43].

### 4.3. Construction of Gene-Deletion Strains

The plasmid and PCR products used in this study are shown in Table 2, and the PCR primers used are listed in Table 3. A multiple gene knockout system with one-step PCR (MONSTER) [43] was utilized to prepare approximately 2.5 kb knockout cassettes (MONSTER-rad55, MONSTER-alhr3, MONSTER-_Saci_0790, and MONSTER-sftH, respectively) and to construct the *rad55*-, *alhr3*-, Saci_0790-, and *sftH*-deletion strains. In brief, MONSTER-rad55 was amplified from placSpyrE via PCR using primers MONSTER-rad55-F/R (comprising a 40-bp 5′-flanking region and a 30-bp 3′-flanking region of the *rad55* and a sequence that anneals with the *lacS*-marker gene, and a 40-bp region of *rad55* as a target gene (Tg)-arm and a sequence that anneals with the *pyrE*-marker gene, respectively) and Premix Taq (Ex Taq Version 2.0; Takara Bio, Kusatsu, Japan). Similarly, MONSTER-alhr3, MONSTER-_Saci_0790, and MONSTER-sftH were amplified using each MONSTER-F/R primer set. The purified PCR products (250–800 ng/μL in 5 M Tris–HCl, pH 8.5) were used for subsequent electro-transformation.

The transformation protocol for *S. acidocaldarius* has been previously described [43]. To disrupt *rad55*, 2 μg of MONSTER-rad55 (250 ng/μL) was electroporated into DP-1 cells that were harvested at the late-log phase (OD_600_ = 0.538). After electroporation (15 kV/cm, 9 mS), the cells were spread on a selective uracil-free XT plate. After cultivation at 75 °C for 6 d, the colonies that were grown on the plate were treated with an X-gal solution (10 mg/mL) and further cultivated at 75 °C for 1 d. Transformants that formed blue colonies were selected, and the genomic DNA was analyzed via PCR using the outer primer set rad55-out-F/R to detect the insertion of MONSTER-rad55. This intermediate strain was named DP-13 Int (*pyrE*^+^ *lacS*^+^) and was used for pop-out recombination. After cultivation on XTUF plates, followed by X-gal visualization, white colonies were selected and analyzed by colony PCR using outer primer sets. The *rad55* gene disruptant (Δ*pyrE* Δ*suaI* Δ*phr* Δ*rad55*) was designated DP-13. Similarly, DP-14 (Δ*pyrE* Δ*suaI* Δ*phr* Δ*alhr3*), DP-16 (Δ*pyrE* Δ*suaI* Δ*phr* ΔSaci_0790), and DP-18 (Δ*pyrE* Δ*suaI* Δ*phr* Δ*sftH*) were constructed (Figure S1). Regarding the purification of DP-16 Int, we performed dilution-to-extinction using a liquid medium but did not perform single colony isolation on the plates. Strains DP-5 and DP-17 were previously constructed as the *ssb*- and *alhr1*-deletion strains, respectively [37,38], and were used in this study.

### 4.4. Construction of the pyrE-Proficient Strain

The procedure for the construction of the *pyrE*-proficient strain was previously described [17]. The *pyrE*-proficient strains DP-1 *pry*^+^ (Δ*suaI* Δ*phr*) and DP-11 (Δ*suaI* Δ*phr* Δ*ssb*) were constructed from the parental strain DP-1 and the *ssb*-deleted strain DP-5, respectively, by the complementation of the *pyrE* gene.

### 4.5. Estimation of the Mutation Rate

To estimate the mutation rate, 200 µL of each stationary phase culture of the *pyrE* proficient strain DP-1 *pry*^+^ (OD_600_ = 0.845) and DP-11 (OD_600_ = 0.782) was spread on XTUF plates, which were then incubated at 75 °C for 5–7 d. To investigate the impact of the pre-cultivation temperature of DP-11 on the mutation rate, each culture of DP-1 *pry*^+^ and DP-11, cultivated at 60 °C, was used as an inoculum for plating. The colonies that appeared on the plate were scored, and the mutation frequency (colonies on XTUF plate/plating 10^8^ cells) was calculated. The total plating cell number was calculated from the cell density of 3.4 × 10^8^/mL (OD_600_ = 1). The experiments were repeated in triplicate using the same culture.

### 4.6. Mating Test

In addition to the uracil-auxotrophic strains DP-1 and DP-5 based on MR31 [55], which contain a 31-bp deletion in *pyrE*, three uracil-auxotrophic strains (*pyr*^–^) were prepared from the *pyrEF*-proficient strains DP-1 *pry*^+^ (*ssb*^+^) and DP-11 (Δ*ssb*) under negative selection on an XYUF plate followed by the isolation of the colonies that appeared. The resulting uracil-auxotrophic parental strain DP-2 (*ssb*^+^) and the *ssb*-deleted strains DP-11-1 (Δ*ssb*) and DP-11-3 (Δ*ssb*) were used for the mating test.

To assay the DNA exchange between *S. Acidocaldarius* cells, a mating test [56] was performed. For the mating test, each log phase of culture (5 × 10^6^ cells) for DP-1, DP-2, DP-5, DP-11-1, and DP-11-3 cultivated at 60 or 75 °C on a block heater was plated on an XT plate as a negative control. The recombination between the strains, as indicated above, was performed by mixing different combinations of two strains on XT plates to select recombination events by spreading 2.5 × 10^6^ cells per strain. The plate was incubated at 75 °C for 6 days. The resulting colonies (uracil-proficient colonies) were counted as recombinant colonies. To calculate the actual number of recombinant colonies, the number of false positive colonies from the negative control was subtracted.

### 4.7. Estimation of HR Frequencies

The estimation of HR frequencies was carried out as reported previously [43]. Between two hundred and two hundred fifty nanograms of a linear marker cassette, pyrElacS800, harboring approximately 0.8-kb 5′- and 3′-flanking regions of the *suaI* locus attached to both ends of the *pyrE*-*lacS* marker [43] (Table 2), was electroporated (15 kV/cm and 9 ms) into each competent cell, and the samples were plated on XT plates. The plates were incubated at 75 °C for 6–7 days. The colonies that appeared were counted. As a control experiment, an autonomously replicating plasmid vector pSAV2 containing the *pyrE* selectable marker [54] was used to calculate the transformation efficiency of each strain. The HR frequency and transformation efficiency were defined as the number of transformants per 1 μg DNA for both assays.

### 4.8. Growth Curve after UV Irradiation

The growth curve procedure after UV-B irradiation has been previously described [45]. One milliliter of each overnight culture (late-log to stationary phase) was poured into 90 × 15 mm plastic Petri dishes (AGC TECHNO GLASS, Yoshida-cho, Shizuoka, Japan) and then exposed to UV-B irradiation using a UV lamp (UVM-57, 302 nm, 6 W; Analytik Jena AG Jena, Germany) positioned 6.5 cm above the top of the dish at room temperature for zero, 40, and 60 s (yielding the expected zero, 800, and 1200 J/m^2^, respectively). An irradiated sample was inoculated in 6 mL of the XTU liquid medium to yield an initial OD_600_ = 0.005 (in triplicate). The cells were then cultivated at 75 °C with shaking at 160 rpm. Thereafter, cell growth was monitored by measuring the OD_600_.

To examine the effects of the pre-and post-cultivation temperature on UV sensitivity, cultures of Δ*ssb* (DP-5) cultivated at 60 or 75 °C were used for UV exposure. Then, irradiated samples were inoculated in a 6 mL XTU liquid medium to yield an initial OD_600_ = 0.005 by calculation (duplicates). The cells were then cultivated at 60 or 75 °C without shaking on the block heater. Then, the cap of the test tube was loosely opened. Thereafter, cell growth was monitored by measuring the OD_600_.

### 4.9. Analysis of the DNA Repair Properties of CPDs

The method for assaying the DNA repair properties of CPDs has been previously described [45].

### 4.10. Growth Curve in the Presence of DNA-Damaging Agents

The procedure for generating the growth curves for strains in the presence of DNA-damaging agents (cisplatin, metronidazole, and 4-nitroquinoline N-oxide [4-NQO]) has been previously described [45].

### 4.11. UV, H_2_O_2_, and Heat-Shock Survival Tests Using a Spotting Assay

Procedures for the UV-B, hydrogen peroxide (H_2_O_2_), and heat-shock survival tests using a spotting assay have been previously described [17,37].

### 4.12. Growth Curve at Various Temperatures

To characterize the range of growth temperatures, each overnight culture (stationary phase) was inoculated in 6 mL of the XTU liquid medium to yield an initial OD_600_ = 0.005. Inoculation was performed in triplicate using the same overnight culture. The cells were then cultivated at 50–80 °C (temperature range from minimal to maximal growth temperature) with 5 °C intervals without shaking on the block heater. Then, the cap of the test tube was loosely opened. Thereafter, cell growth was monitored by measuring the OD_600_.

### 4.13. Analysis of the Distribution of the Gene Saci_0790

The distribution of the Saci_0790 homologs in Archaea was searched in the NCBI OrthoDB catalog using a protein sequence of Saci_0790.

## Figures and Tables

**Table 1:** Sensitivities of gene-deleted strains to DNA damage.

	Type of DNA Damage	DP-1	Δ*ssb*	Δ*alhr1*	Δ*alhr3*	Δ*sftH*	Δ*rad55*	ΔSaci_0790
UV	CPD	−	+	+	±	−	±	+ +
Cisplatin	Intra-strand Crosslink ^a^	−	+ +	+	− −	+	+	+ +
4-NQNO	Bulky adduct ^a^	−	+	+ +	−	−	−	+ +
Metronidazole	Bulky adduct ^a^	−	+	+ +	−	−	−	+ +
H_2_O_2_	Oxidative stress, DSB ^b^	−	+ +	+ +	−	+ +	−	+ +

Sensitivities of gene-deleted strains to DNA damage are summarized. − −, −, ±, +, and + + indicate tolerant, no sensitivity, slightly sensitive, sensitive, and markedly sensitive, respectively. No sensitivity means that the sensitivity of the gene-deleted strain is the same as that of the parental strain. Type of DNA damage is cited from ^a^ Sakofsky et al. [51] and ^b^ Imlay et al. [46].

**Table 2:** Strains or DNAs used in this study.

Strains or DNAs	Relevant Characteristic(s)	Source or Reference
Strains		
*S.acidocaldarius*		
DP-1	SK-1 with Δ*phr* (Δ*pyrE* Δ*suaI* Δ*phr*)	[43]
DP-1*pyr*^+^	*pyrE*^+^ strain derivative from DP-1 (Δ*suaI* Δ*phr*)	This study
DP-2	*pyr^–^* strain derivative from DP-1 *pyr*^+^ (*pyr*^–^ Δ*suaI* Δ*phr*)	This study
DP-5	DP-1 with Δ*ssb* (Δ*pyrE* Δ*suaI* Δ*phr* Δ*ssb*)	[37]
DP-11	*pyrE*^+^ strain derivative from DP-5 (Δ*suaI* Δ*phr* Δ*ssb*)	This study
DP-11-1	*pyr*– strain derivative from DP-5 (*pyr*^–^ Δ*suaI* Δ*phr* Δ*ssb*)	This study
DP-11-3	*pyr*– strain derivative from DP-5 (*pyr*^–^ Δ*suaI* Δ*phr* Δ*ssb*)	This study
DP-13	DP-1 with Δ*rad*55 (Δ*pyrE* Δ*suaI* Δ*phr* Δ*rad*55)	This study
DP-14	DP-1 with Δ*alhr3* (Δ*pyrE* Δ*suaI* Δ*phr* Δ*alhr3*)	This study
DP-16	DP-1 with ΔSaci_0790 (Δ*pyrE* Δ*suaI* Δ*phr* ΔSaci_0790)	This study
DP-17	DP-1 with Δ*alhr1* (Δ*pyrE* Δ*suaI* Δ*phr* Δ*alhr1*)	[38]
DP-18	DP-1 with Δ*sftH* (Δ*pyrE* Δ*suaI* Δ*phr* Δ*sftH*)	This study
Plasmid		
placSpyrE	Plasmid DNA carrying 0.8 kb of 5′ and 3′ flanking regions of *suaI* locus at both ends of *pyrE*-*lacS* dual marker	[43]
pSAV2	*Sulfolobus*-*E. coli* shuttle vector, based on pBluescript II KS (−) and pRN1, with the *SsopyrEF* maker	[54]
PCRproducts		
MONSTER-rad55	Linear DNA containing the 40 bp 5′ and 30 bp 3′ flanking regions of *rad55*, and a 40 bp region of *rad55* as the Tg-arm at both ends of *pyrE*-*lacS* dual marker	This study
MONSTER-Saci_0790	Linear DNA containing the 40 bp 5′ and 30 bp 3′ flanking regions of Saci_0790, and a 40 bp region of Saci_0790 as the Tg-arm at both ends of *pyrE*-*lacS* dual marker	This study
MONSTER-alhr3	Linear DNA containing the 40 bp 5′ and 30 bp 3′ flanking regions of *alhr3*, and a 40 bp region of *alhr3* as the Tg-arm at both ends of *pyrE*-*lacS* dual marker	This study
MONSTER-sftH	Linear DNA containing the 39 bp 5′ and 30 bp 3′ flanking regions of *sftH*, and a 39 bp region of *sftH* as the Tg-arm at both ends of *pyrE*-*lacS* dual marker	This study
pyrElacS800	Linear DNA carrying 0.8 kb of 5′ and 3′ flanking regions of *suaI* locus at both ends of *pyrE*-*lacS* dual marker	[43]

**Table 3:** Primers used in this study.

Primers	Sequence ^a^ (5′-3′)
MONSTER-rad55-F	tcatctgtgtttttaatgtaacaagagttaatataaattt**aaaaagtaatggataaaattaaggaagctg**TTTTTCTCTATATCAATCTC
MONSTER-rad55-R	gcttgtcgaactcatatatacctgttgataatcttatcacTCCTAGATCTAAAACTAAAG
rad55-out-F	catcctgtgtataaggaatg
rad55-out-R	atatgcagaaactggtgttg
MONSTER-alhr3-F	atatccgtttaatgtgcattgaacatatccggtggtatat**tgaggccctttcaatagattggtgataaag**TTTTTCTCTATATCAATCTC
MONSTER-alhr3-R	ttagtgatagtagcttgtagctaagatcatttttatccacTCCTAGATCTAAAACTAAAG
alhr3-out-F	ttactgttattttgattccttg
alhr3-out-R	tagatttggtaataacgatttc
MONSTER-Saci_0790-F	taactaattttttaatacaaaggagaagagtatttagtga**gaaaacttgtggaagaaggattggcaatct**TTTTTCTCTATATCAATCTC
MONSTER-Saci_0790-R	tatattcttcttcagataactttatataaatggtcttcatTCCTAGATCTAAAACTAAAG
Saci_0790-out-F	tttataggagtaccttatgag
Saci_0790-out-R	atctttgccaggacattaac
MONSTER-sftH-F	gtaataaaattgtccactgaattaattgatagagtttca**aaacttggtgaatttgataattcggttgaa**GTTTTTCTCTATATCAATCTC
MONSTER-sftH-R	tatgtgggcaatcttgacgttaaaatacgataacctctcCTCCTAGATCTAAAACTAAAG
sftH-out-F	cttctcgatttccttataattg
sftH-out-R	cgtacttgacaacagtaaag
SAMR31-F	gatttcgtgaaagctctacttg
SAMR31-R	tttttctcagctctgatgtatc

^a^ 5′ homologous regions of the target gene are underlined with a solid line, that of 3′ is in **bold**, Tg-arm is underlined with a dotted line, and sequences of MONSTER primers that anneal with the *pyrE*-*lacS* dual marker gene are in capital letters.

## Data Availability

Not applicable.

## Supplementary Materials

The following supporting information can be downloaded at: https://www.mdpi.com/article/10.3390/ijms24054558/s1.

## References

[1] Lindahl T. (1993). Instability and decay of the primary structure of DNA. Nature.

[2] Stetter K.O. (2006). Hyperthermophiles in the history of life. Philos. Trans. R. Soc. Lond. B Biol. Sci..

[3] Gorgan D.W. (1998). Hyperthermophiles and the problem of DNA stability. Mol. Microbiol..

[4] Grogan D.W. (2000). The question of DNA repair in hyperthermophilic archaea. Trends Microbiol..

[5] Grogan D.W. (2004). Stability and repair of DNA in hyperthermophilic archaea. Mol. Biol..

[6] Grogan D.W. (2015). Understanding DNA repair in hyperthermophilic archaea: Persistent gaps and other reactions to focus on the fork. Archaea.

[7] Ishino Y., Nishino T., Morikawa K. (2006). Mechanisms of Maintaining Genetic Stability by Homologous Recombination. Chem. Rev..

[8] White M.F. (2011). Homologous recombination in the archaea: The means justify the ends. Biochem. Soc. Trans..

[9] Grasso S., Tell G. (2014). Base excision repair in Archaea: Back to the future in DNA repair. DNA Repair.

[10] Ishino Y., Narumi I. (2015). DNA repair in hyperthermophilic and hyperradioresistant microorganisms. Curr. Opin. Microbiol..

[11] White M.F., Allers T. (2018). DNA repair in the archaea—An emerging picture. FEMS Microbiol. Rev..

[12] Craig J., Marshall C.J., Santangelo T.J. (2020). Archaeal DNA Repair Mechanisms. Biomolecules.

[13] White M.F., Garrett R.A., Klenk H.P. (2007). DNA repair. Archaea: Evolution, Physiology and Molecular Biology.

[14] Rouillon C., White M.F. (2011). The evolution and mechanisms of nucleotide excision repair proteins. Res. Microbiol..

[15] Fujikane R., Ishino S., Ishino Y., Forterre P. (2010). Genetic analysis of DNA repair in the hyperthermophilic archaeon, *Thermococcus kodakaraensis*. Genes Genet. Syst..

[16] Zhang C., Tian B., Li S., Ao X., Dalgaard K., Gökce S., Liang Y., She Q. (2013). Genetic manipulation in *Sulfolobus islandicus* and functional analysis of DNA repair genes. Biochem. Soc. Trans..

[17] Suzuki S., Kurosawa N. (2019). Endonucleases responsible for DNA repair of helix-distorting DNA lesions in the thermophilic crenarchaeon *Sulfolobus acidocaldarius* in vivo. Extremophiles.

[18] Komori K., Fujikane R., Shinagawa H., Ishino Y. (2002). Novel endonuclease in archaea cleaving with various branched structure. Genes Genet. Syst..

[19] Roberts J.A., Bell S.D., White M.F. (2003). An archaeal XPF repair endonuclease dependent on a heterotrimeric PCNA. Mol. Microbiol..

[20] Roberts J.A., White M.F. (2005). An archaeal endonuclease displays key properties of both eukaryal XPF-ERCC1 and Mus81. J. Biol. Chem..

[21] Ren B., Kühn J., Meslet-Cladiere L., Briffotaux J., Norais C., Lavigne R., Flament D., Ladenstein R., Myllykallio H. (2009). Structure and function of a novel endonuclease acting on branched DNA substrates. EMBO J..

[22] Ishino S., Nishi Y., Oda S., Uemori T., Sagara T., Takatsu N., Yamagami T., Shirai T., Ishino Y. (2016). Identification of a mismatch-specific endonuclease in hyperthemophilic archaea. Nucleic Acids Res..

[23] Ahmad S., Huang Q., Ni J., Xiao Y., Yang Y., Shen Y. (2020). Functional analysis of the NucS/EndoMS of the hyperthermophilic archaeon *Sulfolobus islandicus* REY15A. Front. Microbiol..

[24] Murzin A.G. (1993). OB(oligonucleotide/oligosaccharide binding)-fold: Common structural and functional solution for non-homologous sequences. EMBO J..

[25] Chédin F., Seitz E.M., Kowalczykowski S.C. (1998). Novel homologs of replication protein A in Archaea: Implications of the evolution of ssDNA-binding proteins. Trends Biochem. Sci..

[26] Kerr I.D., Wadsworth R.I., Cubeddu L., Blankenfeldt W., Naismith J.H., White M. (2003). Insights into ssDNA recognition by the OB fold from a structural and thermodynamic study of Sulfolobus SSB protein. EMBO J..

[27] Oliveira M.T. (2021). Single Stranded DNA Binding Proteins.

[28] Paytubi S., McMahon S.A., Graham S., Liu H., Botting C.H., Makarova K.S., Koonin E.V., Naismith J.H., White M.F. (2012). Displacement of the canonical single-stranded DNA-binding protein in the Thermoproteales. Proc. Natl. Acad. Sci. USA.

[29] Meyer R.R., Glassberg J., Kornberg A. (1979). An *Escherichia coli* mutant defective in single-strand binding protein is defective in DNA replication. Proc. Natl. Acad. Sci. USA.

[30] Glassberg J., Meyer R.R., Kornberg A. (1979). Mutant single-strand binding protein of *Escherichia coli*: Genetic and physiological characterization. J. Bacteriol..

[31] Longhese M.P., Plevani P., Lucchini G. (1994). Replication factor A is required in vivo for DNA replication, repair, and recombination. Mol. Cell. Biol..

[32] Muniyappa K., Shaner S.L., Tsang S.S., Radding C.M. (1984). Mechanism of the concerted action of recA protein and helix-destabilizing proteins in homologous recombination. Proc. Natl. Acad. Sci. USA.

[33] Sugiyama T., Zaitseva E.M., Kowalczykowski S.C. (1997). A single-stranded DNA-binding protein is needed for efficient presynaptic complex formation by the *Saccharomyces cerevisiae* Rad51 protein. J. Biol. Chem..

[34] Komori K., Ishino Y. (2001). Replication Protein A in *Pyrococcus furiosus* Is Involved in Homologous DNA Recombination. J. Biol. Chem..

[35] Haseltine C.A., Kowalczykowski S.C. (2002). A distinctive single-stranded DNA-binding protein from the Archaeon *Sulfolobus solfataricus*. Mol. Microbiol..

[36] Cubeddu L., White M.F. (2005). DNA Damage Detection by an Archaeal Single-stranded DNA-binding Protein. J. Mol. Biol..

[37] Suzuki S., Kurosawa N. (2019). Robust growth of archaeal cells lacking a canonical single-stranded DNA-binding protein. FEMS Microbiol. Let..

[38] Suzuki S., Kurosawa N., Yamagami T., Matsumoto S., Numata T., Ishino S., Ishino Y. (2022). Genetic and Biochemical Characterizations of aLhr1 Helicase in the Thermophilic Crenarchaeon *Sulfolobus acidocaldarius*. Catalysts.

[39] Chamieh H., Ibrahim H., Kozah J. (2016). Genome-wide identification of SF1 and SF2 helicases from archaea. Gene.

[40] Hajj M., Langendijk-Genevaux P., Batista M., Quentin Y., Laurent S., Abdel Razzak Z., Flament D., Chamieh H., Fichant G., Clouet-d’Orval B. (2021). Phylogenetic Diversity of Lhr Proteins and Biochemical Activities of the *Thermococcales* aLhr2 DNA/RNA Helicase. Biomolecules.

[41] McRobbie A.M., Carter L.G., Kerou M., Liu H., McMahon S.A., Johnson K.A., Oke M., Naismith J.H., White M.F. (2009). Structural and functional characterization of a conserved archaeal RadA paralog with antirecombinase activity. J. Mol Biol..

[42] Liang P.J., Han W.Y., Huang Q.H., Li Y.Z., Ni J.F., She Q.X., Shen Y.L. (2013). Knockouts of RecA-like proteins RadC1 and RadC2 have distinct responses to DNA damage agents in *Sulfolobus islandicus*. J. Genet. Genom..

[43] Suzuki S., Kurosawa N. (2017). Development of the multiple gene knockout system with one-step PCR in thermophilic crenarchaeon *Sulfolobus acidocaldarius*. Archaea.

[44] Grogan D.W. (1996). Exchange of genetic markers at extremely high temperatures in the archaeon *Sulfolobus acidocaldarius*. J. Bacteriol..

[45] Suzuki S., Kurosawa N. (2019). Participation of UV-regulated genes in the response to helix-distorting DNA damage in the thermoacidophilic crenarchaeon *Sulfolobus acidocaldarius*. Microbes Environ..

[46] Imlay J.A., Chin S.M., Linn S. (1988). Toxic DNA damage by hydrogen peroxide through the Fenton reaction in vivo and in vitro. Science.

[47] Ariyoshi M., Morikawa K. (2016). A dual base flipping mechanism for archaeal mismatch repair. Structure.

[48] Nakane S., Hijikata A., Yonezawa K., Kouyama K., Mayanagi K., Ishino S., Ishino Y., Shirai T. (2016). Structure of the EndoMS-DNA complex as mismatch restriction endonuclease. Structure.

[49] Miyabayashi H., Sakai D.H., Kurosawa N. (2021). DNA polymerase B1 binding protein 1 is important for DNA repair by holoenzyme PolB1 in the extremely thermophilic crenarchaeon *Sulfolobus acidocaldarius*. Microorganisms.

[50] Bell G.D., Grogan D.W. (2002). Loss of genetic accuracy in mutants of the thermoacidophile *Sulfolobus acidocaldarius*. Archaea.

[51] Sakofsky C.J., Runck L.A., Grogan D.W. (2011). *Sulfolobus* mutants, generated via PCR products, which lack putative enzymes of UV photoproduct repair. Archaea.

[52] Song X., Huang Q., Ni J., Yu Y., Shen Y. (2016). Knockout and functional analysis of two DExD/H-box family helicase genes in *Sulfolobus islandicus* REY15A. Extremophiles.

[53] Valenti A., Napoli A., Ferrara M.C., Nadal M., Rossi M., Ciaramella M. (2006). Selective degradation of reverse gyrase and DNA fragmentation induced by alkylating agent in the archaeon *Sulfolobus solfataricus*. Nucleic Acids Res..

[54] Suzuki S., Kurosawa N. (2016). Disruption of the gene encoding restriction endonuclease SuaI and development of a host–vector system for the thermoacidophilic archaeon *Sulfolobus acidocaldarius*. Extremophiles.

[55] Reilly M.S., Grogan D.W. (2001). Characterization of intragenic recombination in a hyperthermophilic archaeon via conjugational DNA exchange. J. Bacteriol..

[56] Van Wolferen M., Ma X., Albers S.-V. (2015). DNA Processing Proteins Involved in the UV-Induced Stress Response of *Sulfolobales*. J. Bacteriol..

