# Supplementary material for: The Impact of Single-Stranded DNA-Binding Protein SSB and Putative SSB-Interacting Proteins on Genome Integrity in the Thermophilic Crenarchaeon Sulfolobus acidocaldarius
Source: Int J Mol Sci. 2023 Feb 25;24(5):4558. doi: 10.3390/ijms24054558 (PMC10003305; doi:10.3390/ijms24054558)
Supplement: Supplementary file 1 [file ijms-24-04558-s001.zip › ijms-2206454-supplementary.pdf]

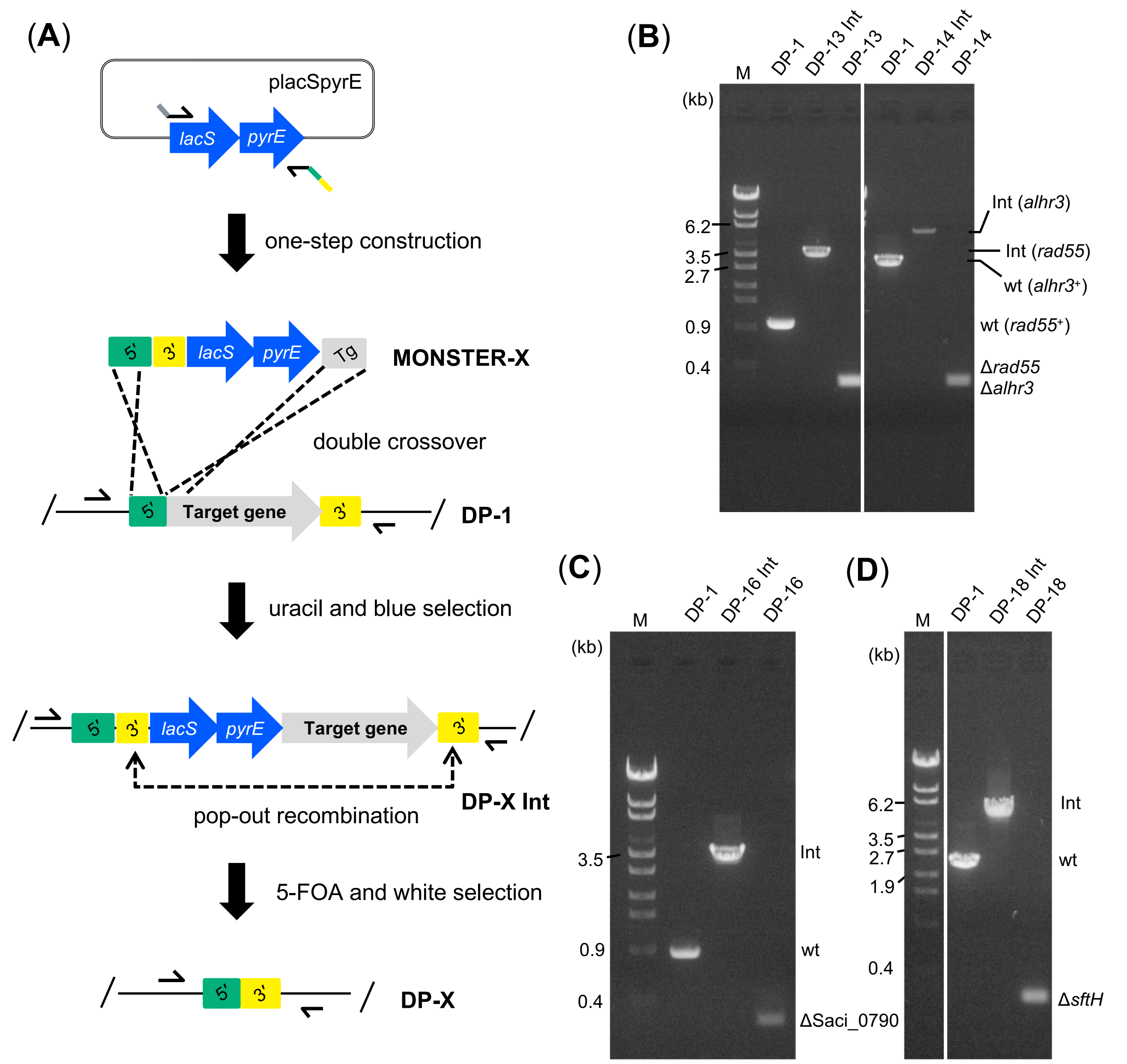

Figure S1. Construction of gene-deleted strains using MONSTER. (**A**) Schematic drawing of the construction of a gene-deletion mutant. The MONSTER-X (X = *rad55*, *alhr3*, Saci_0790, and *sftH*) cassette was amplified and electroporated into the DP-1 strain. A double crossover between MONSTER-X and the chromosome at the 5′-flanking and Tg regions resulted in the insertion of the *lacS-pyrE* marker and the 3′-flanking region at a target gene locus. Transformants forming blue colonies were selected on uracil-free plates. The excision of target and counterselectable marker genes was achieved through pop-out recombination. PCR analyses were performed on genomic DNA using the outer primer set. Arrows show the positions of the outer primer sets. PCR analysis using outer primers is shown in (**B**) *rad55* and *alhr3*, (**C**) Saci_0790, and (**D**) *sftH*. The expected sizes of the products were as follows: (1) *rad55* locus; 1,003 bp (wt), 3,609 bp (Int), and 217 bp (Δ*rad55*). (2) *alhr3* locus; 3,094 bp (wt), 5,610 bp (Int), and 289 bp (Δ*alhr3*). (3) Saci_0790 locus; 913 bp (wt), 3,519 bp (Int), and 275 bp (ΔSaci_0790). (4) *sftH* locus; 2,412 bp (wt), 5,018 bp (Int), and 261 bp (Δ*sftH*). Note: A λ EcoT14 ladder was loaded in lane M.

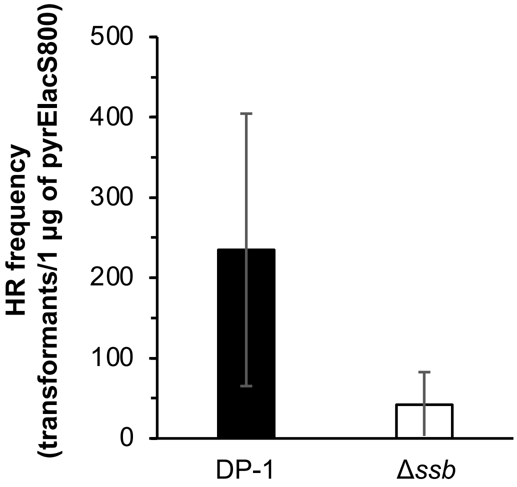

Figure S2. HR frequency of the *ssb*-deleted strain. Transformation of DP-1 and DP-5 (Δ*ssb*) with pyrElacS800 was carried out. Data are presented as mean ±SD calculated from the values of eight independent experiments.

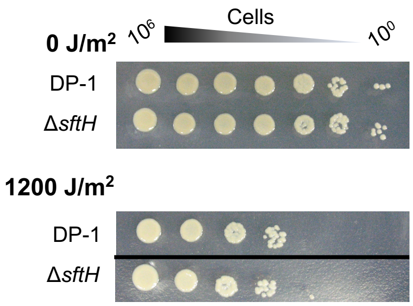

Figure S3. UV survival of the *sftH*-deleted strain. Each overnight culture of DP-1 (parent strain) and Δ*sftH* was exposed to UV-B light (0 and 1200 J/m^2), and aliquots were serially diluted (10^0–10^{-6} corresponding to 10^6–10^0 cells) and spotted onto plates. Plates were incubated at 75°C. Experiments were repeated three times with similar results.

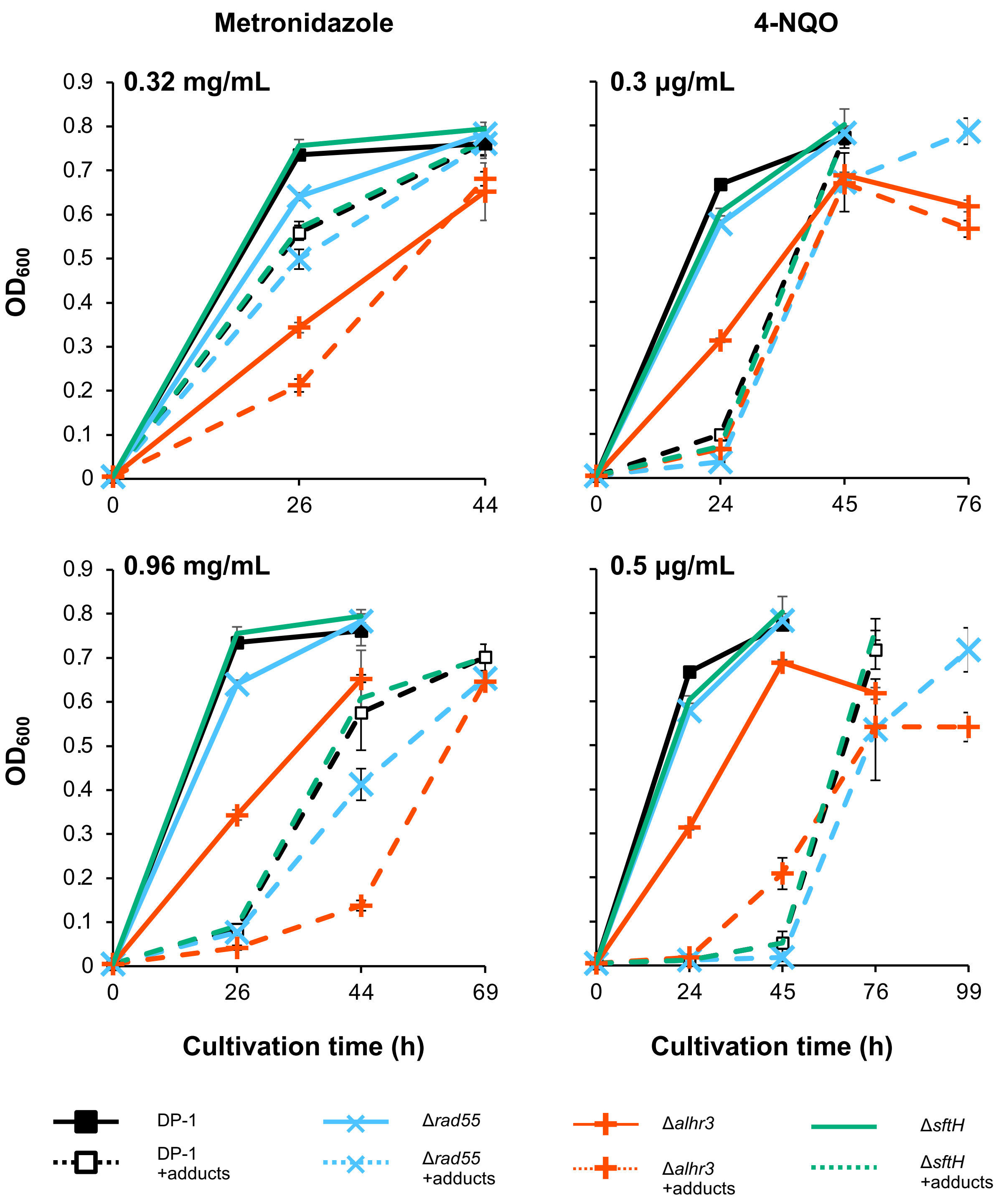

Figure S4. Growth curve of Δ*rad55*, Δ*alhr3*, and Δ*sftH* in the presence of metronidazole and 4-NQO. Each overnight culture of DP-1 (parent strain), Δ*rad55*, Δ*alhr3*, and Δ*sftH* was inoculated in liquid medium in the presence of metronidazole (0.32 and 0.96 mg/mL) and 4-NQO (0.3 and 0.5 µg/mL), and cultivated at 75°C with shaking. Solid and dotted (+adducts) lines indicate growth curves in the absence or presence of DNA adducts, respectively. Error bars represent ±SD calculated using three biological replicates.

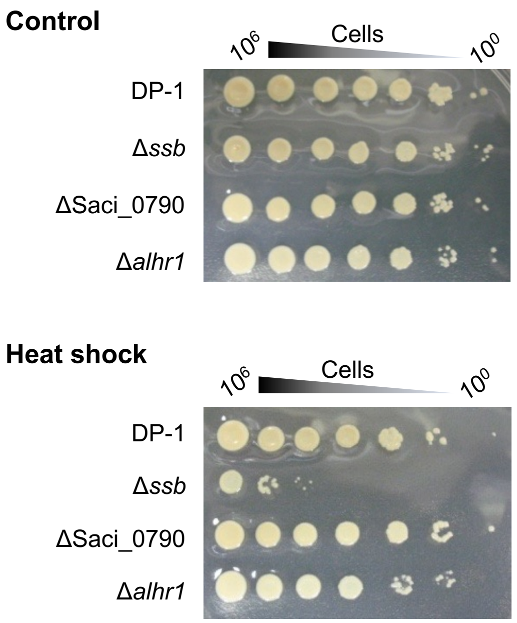

Figure S5. Heat-shock test of gene-deleted strains. After each of the cultures was treated with heat shock at 90°C for 3 min followed by preparation of diluted samples (10^0–10^{-6} corresponding to 10^6–10^0 cells), the samples were spotted onto plates and cultivated at 75°C. Controls indicate mock-treated samples. Experiments were repeated three times with similar results.

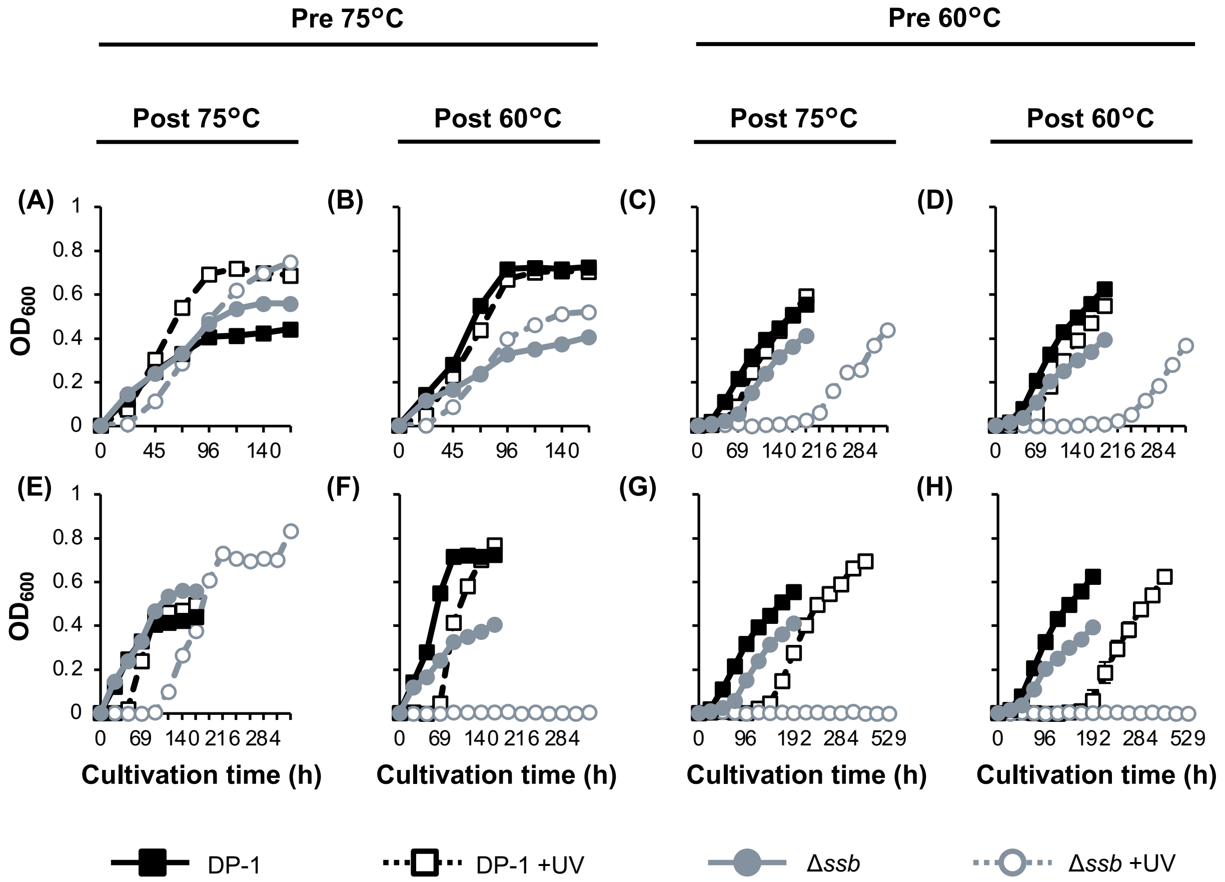

Figure S6. Significant sensitivity of the *ssb*-deleted strain to UV irradiation. Each culture of DP-1 and Δ*ssb* that was cultivated at 75°C (**A**, **B**, **E**, and **F**) and 60°C (**C**, **D**, **G**, and **H**) (precultivation temperature denoted by pre 75°C and 60°C, respectively) was irradiated with UV-B light (600 [**A**–**D**] and 1200 J/m^2 [**E**–**F**]), inoculated in liquid medium and incubated at 75°C (**A**, **C**, **E**, and **G**) and 60°C (**B**, **D**, **F**, **H**) (postcultivation temperature denoted by post 75°C and 60°C, respectively) without shaking. Solid and dotted (+adducts) lines indicate growth curves of samples treated without or with UV irradiation, respectively. (n = 2).

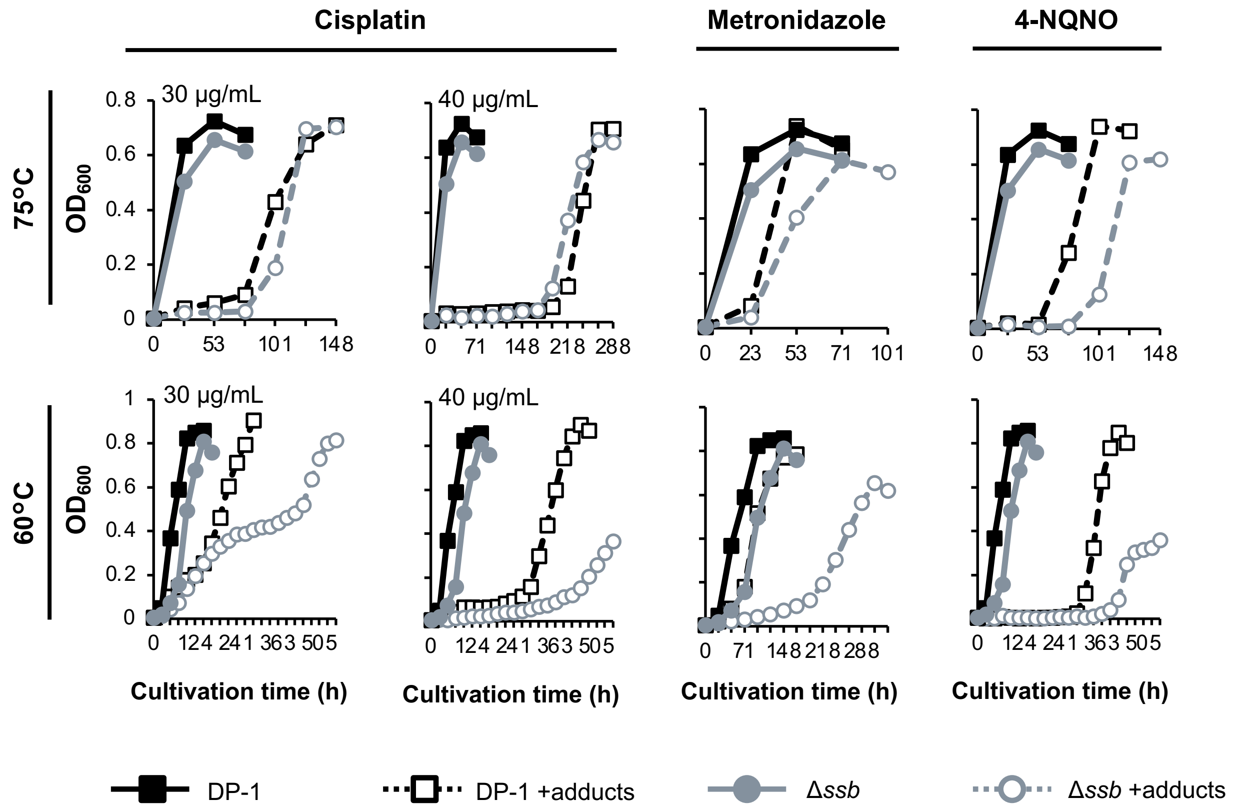

Figure S7. Significant sensitivity of the *ssb*-deleted strain to DNA adducts at lower temperature. Each overnight culture of DP-1 and Δ*ssb* was inoculated in liquid medium in the presence of DNA adducts (cisplatin [30 and 40 μg/mL], metronidazole [1.2 mg/mL], 4-NQNO [0.5 μg/mL]) and cultivated at 60°C (lower growth curves) and 75°C (upper growth curves) without shaking. Solid and dotted (+adducts) lines indicate growth curves in the absence or presence of DNA adducts, respectively. (n = 2).
